# A cell competition–based small molecule screen identifies a novel compound that induces dual c-Myc depletion and p53 activation

**DOI:** 10.1074/jbc.RA120.015285

**Published:** 2020-12-17

**Authors:** Dagim Shiferaw Tadele, Joseph Robertson, Richard Crispin, Maria C. Herrera, Markéta Chlubnová, Laure Piechaczyk, Pilar Ayuda-Durán, Sachin Kumar Singh, Tobias Gedde-Dahl, Yngvar Fløisand, Jørn Skavland, Jørgen Wesche, Bjørn-Tore Gjertsen, Jorrit M. Enserink

**Affiliations:** 1 Department of Molecular Cell Biology, Institute for Cancer Research, The Norwegian Radium Hospital, Oslo, Norway; 2 Section for Biochemistry and Molecular Biology, Faculty of Mathematics and Natural Sciences, University of Oslo, Oslo, Norway; 3 Department of Tumor Biology, Institute for Cancer Research, The Norwegian Radium Hospital, Oslo, Norway; 4 Department of Hematology, Oslo University Hospital, Oslo, Norway; 5 Precision Oncology Research Group, Department of Clinical Science, University of Bergen, Bergen, Norway

**Keywords:** c-Myc, p53, drug screening, anticancer drug, mRNA, phosphoproteomics, Abl, tyrosine kinase, leukemia, ALL, acute lymphoid leukemia, AML, acute myelocytic leukemia, CML, chronic myeloid leukemia, DMSO, dimethyl sulfoxide, EGFR, epidermal growth factor receptor, IL3, interleukin-3, LSCs, leukemic stem cells, Ph+, Philadelphia chromosome–positive, PRIDE, Proteomics IDEntifications Database, qPCR, quantitative PCR, SCF, Skp, Cullin, F-box, TKIs, tyrosine kinase inhibitors

## Abstract

Breakpoint Cluster Region-Abelson kinase (BCR–Abl) is a driver oncogene that causes chronic myeloid leukemia and a subset of acute lymphoid leukemias. Although tyrosine kinase inhibitors provide an effective treatment for these diseases, they generally do not kill leukemic stem cells (LSCs), the cancer-initiating cells that compete with normal hematopoietic stem cells for the bone marrow niche. New strategies to target cancers driven by BCR–Abl are therefore urgently needed. We performed a small molecule screen based on competition between isogenic untransformed cells and BCR–Abl-transformed cells and identified several compounds that selectively impair the fitness of BCR–Abl-transformed cells. Interestingly, systems-level analysis of one of these novel compounds, DJ34, revealed that it induced depletion of c-Myc and activation of p53. DJ34-mediated c-Myc depletion occurred in a wide range of tumor cell types, including lymphoma, lung, glioblastoma, breast cancer, and several forms of leukemia, with primary LSCs being particularly sensitive to DJ34. Further analyses revealed that DJ34 interferes with c-Myc synthesis at the level of transcription, and we provide data showing that DJ34 is a DNA intercalator and topoisomerase II inhibitor. Physiologically, DJ34 induced apoptosis, cell cycle arrest, and cell differentiation. Taken together, we have identified a novel compound that dually targets c-Myc and p53 in a wide variety of cancers, and with particularly strong activity against LSCs.

Breakpoint Cluster Region-Abelson kinase (BCR–Abl) is the driver mutation for chronic myeloid leukemia (CML) and is also found in 25% to 30% of Philadelphia chromosome–positive (Ph+) adult acute lymphoid leukemia (ALL) (1, 2). CML and Ph+ ALL are effectively treated with tyrosine kinase inhibitors (TKIs), such as imatinib. However, development of TKI resistance remains an issue, survival of Ph+ ALL patients is suboptimal, and new treatment strategies are required (3).

BCR–Abl activates downstream signaling pathways that promote cell survival and proliferation and that inhibit differentiation, such as the Ras–mitogen-activated protein kinase and PI3K–Akt pathways (4). Another critical component of the BCR–Abl network is the transcription factor c-Myc (5, 6), which regulates genes important for proliferation and survival and which is important for many types of hematological and solid cancers (7).

Populations of self-renewing leukemic stem cells (LSCs) generate the bulk of leukemic cells (8). LSCs are relatively resistant to chemotherapy and persist as a potential source of relapse, and drugs that eradicate LSCs may provide durable remission. LSCs do not require BCR–Abl activity and are therefore resistant to imatinib (9). However, they are highly reliant on both c-Myc and p53, and dual targeting of both c-Myc and p53 has been shown to selectively and synergistically eliminate LSCs (6), demonstrating a need for novel compounds that simultaneously inhibit c-Myc and activate p53.

LSCs compete with healthy hematopoietic stem cells for the bone marrow niche, which constitutes a functional vulnerability of primitive leukemia cells (10). However, cell competition is not typically assayed in high-throughput drug screens, and drugs that reduce the competitiveness of cancer cells without directly affecting cell viability are likely to be discarded.

We reasoned that a straightforward cell competition assay, in which healthy cells and isogenic oncogene-expressing cells compete against each other, would efficiently identify novel compounds that selectively target oncogene-transformed cells. We identified several compounds that preferentially inhibit BCR–Abl-transformed cells, including a compound with anti-LSC activity that dually targets c-Myc/p53.

## Results

### High-throughput cell competition drug screen for BCR–Abl-expressing cells

**Figure 1:**
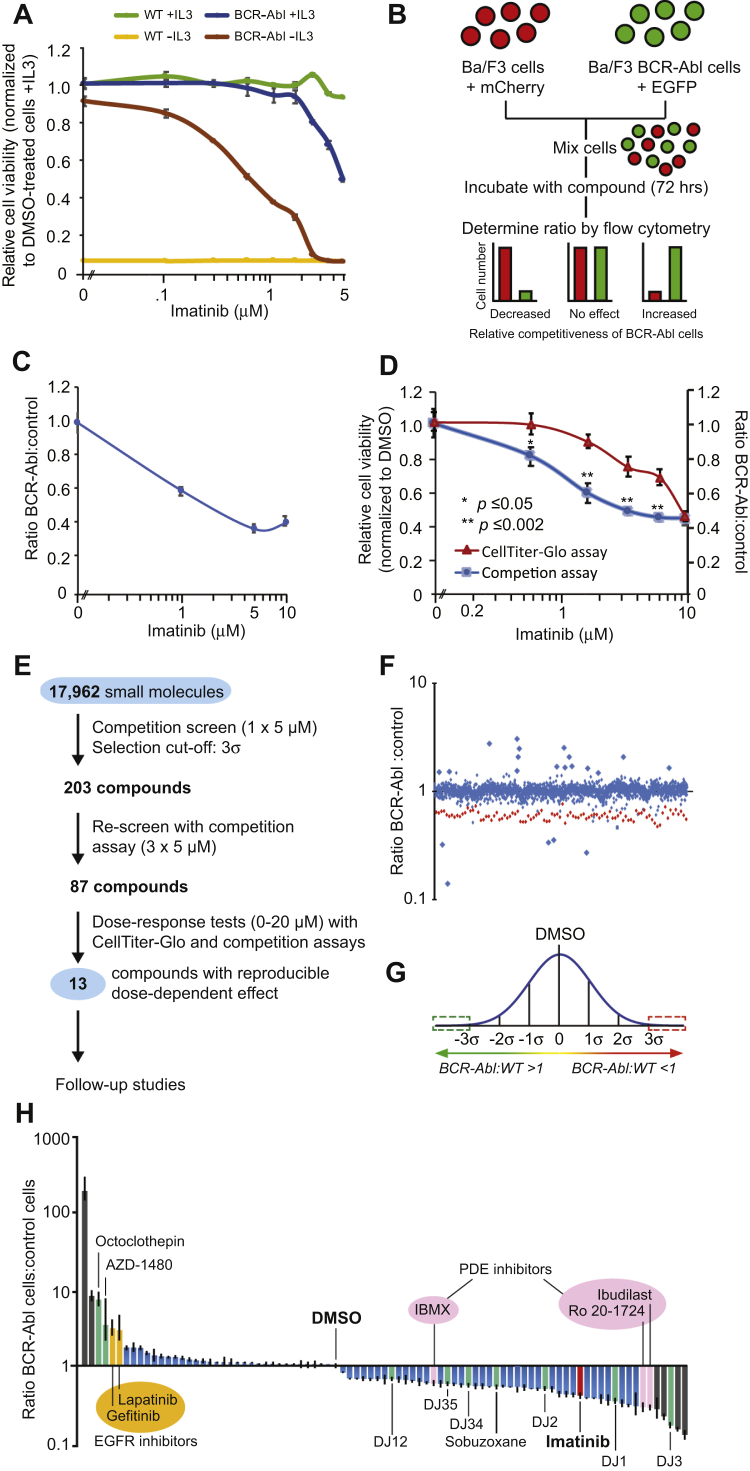
**Development of an isogenic cell competition–based drug screen to identify compounds that modulate the competitiveness of BCR–Abl-expressing cells.***A*, effect of imatinib on the relative viability of WT or BCR–Abl-expressing Ba/F3 cells. Values of WT and BCR–Abl cells were normalized to DMSO treatment in the presence of IL3 for WT and BCR–Abl cells, respectively. *B*, schematic overview of the competition-based drug assay. *C*, ratio of BCR–Abl-expressing cells compared with WT cells following treatment with imatinib for 72 h. *D*, comparison of the selective targeting of BCR–Abl cells by imatinib as measured using a cell viability assay or the competition assay. *E*, schematic overview of the different stages of the competition-based drug screen. *F*, effect of different drugs on the competitiveness of BCR–Abl cells. *Red dots* represent imatinib-treated cells. For display purposes, only a selection of tested compounds is displayed (approximately 20%). *G*, compounds that fell outside the 3σ interval (compared with DMSO-treated cells) were selected for follow-up. *H*, the 87 compounds that were considered for follow-up studies after rescreening of each compound in triplicate but prior to dose–response tests (see panel *E*). All values were normalized to DMSO. *Gray bars* represent compounds that were nonreplicable and therefore removed. *Green bars* represent compounds with a reliable dose-dependent effect, which were selected for follow-up studies; *blue bars* represent compounds with reliable dose-dependent effects but that were not selected for follow-up experiments, mainly because of issues with availability and pricing. Error bars in the relevant panels indicate standard deviation, and statistical significance was determined using Student's *t* test. DMSO, dimethyl sulfoxide; EGFP, enhanced GFP; EGFR, epidermal growth factor receptor; IBMX, isobutylmethylxanthine; IL3, interleukin-3; PDE, phosphodiesterase.

To identify novel compounds that target BCR–Abl-driven leukemias, we developed an isogenic cell competition–based drug screen by stably transfecting Ba/F3 cells with BCR–Abl. Transfection of Ba/F3 cells with BCR–Abl transformed the cells and resulted in interleukin-3 (IL3)–independent growth (Fig. 1*A*). Consistent with previous reports (11, 12), BCR–Abl-transformed cells were significantly more sensitive to imatinib than WT cells, demonstrating they had become oncogene addicted (Fig. 1*A*). Next, BCR–Abl-expressing cells were stably transfected with enhanced GFP and WT cells with mCherry. These cells were mixed and treated with imatinib for 72 h (all experiments involving WT cells were performed in the presence of IL3 unless stated otherwise), after which the BCR–Abl:WT cell ratio was measured by flow cytometry (Fig. 1*B*). Imatinib conferred a competitive disadvantage on BCR–Abl cells (Fig. 1*C*). Importantly, the competition assay was significantly more sensitive than the commonly used CellTiter-Glo cell viability assay (Fig. 1*D*).

**Figure 2:**
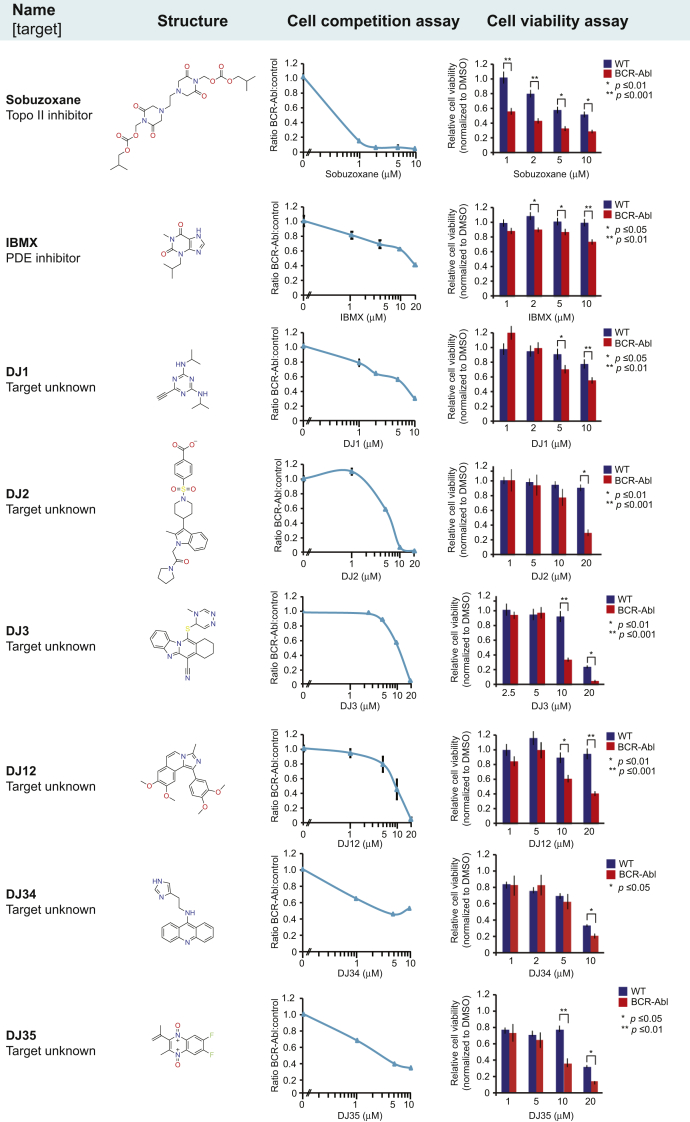
**Eight compounds that decreased the relative competitiveness of BCR–Abl-expressing cells compared with WT cells.** Overview of the names, known molecular targets and structures of the compounds, as well as the results obtained with the cell competition and cell viability assays. All values were normalized to DMSO. Error bars indicate standard deviation, and statistical significance was determined using Student's *t* test. DMSO, dimethyl sulfoxide; IBMX, isobutylmethylxanthine; PDE, phosphodiesterase.

Using the competition assay, we screened 17,962 compounds for selective inhibition of BCR–Abl-expressing cells (Fig. 1*E*). Most compounds had little or no impact on the BCR–Abl:WT cell ratio (Fig. 1*F*). We selected 203 compounds that fell outside the 3σ interval (Fig. 1*G*). These compounds were retested three times, resulting in 87 drugs that significantly (*p* < 0.05) altered the BCR–Abl:WT cell ratio (Fig. 1*H*). This included imatinib, thus validating the screen. All 87 compounds were subjected to dose–response tests with freshly prepared drug stocks using competition assays and viability assays. We found that some compounds did not replicate the effect of the original library compounds (gray bars in Fig. 1*H*). This may have been because of errors during preparation of the original library, contaminants in the initial screen, or compound degradation products. Nonreproducible compounds and compounds that were excessively expensive to synthesize were discarded, leaving a total of 13 compounds that either increased (four compounds, although high doses induced general toxicity; Fig. S1*A*) or decreased (eight compounds; Fig. 2) the competitiveness of BCR–Abl-expressing cells.

### Compounds that increase the competitiveness of BCR–Abl-expressing cells

Among the four compounds that promoted relative competitiveness of BCR–Abl cells was the Janus kinase 2 (JAK2) inhibitor AZD1480 (Fig. S1*A*). Structurally unrelated JAK2 inhibitors had the same effect (Fig. S1*B*), whereas cotreatment with imatinib restored the dependency of BCR–Abl-expressing cells to JAK2 (Fig. S1*C*), consistent with previous studies (13).

The epidermal growth factor receptor (EGFR) inhibitors lapatinib and gefitinib and the G protein–coupled receptor inhibitor octoclothepin also promoted the competitiveness of BCR–Abl cells (Fig. S1*A*), although the reasons for this are currently unclear. Ba/F3 cells do not normally express EGFR, suggesting an off-target effect of EGFR inhibitors, *e.g.*, by inhibiting JAK kinases (14). Octoclothepine inhibits G protein–coupled receptors that elevate intracellular cAMP levels (15), and cAMP impairs survival of BCR–Abl-expressing cells (see later).

### Compounds that reduce the competitiveness of BCR–Abl-expressing cells

We identified eight compounds that reduced the competitiveness of BCR–Abl cells, including sobuzoxane (topoisomerase II inhibitor) and the phosphodiesterase inhibitor isobutylmethylxanthine (Fig. 2). Their effect was validated by unrelated inhibitors (Fig. S2, *A*–*E*). Phosphodiesterase inhibitors increase cellular cAMP levels, which decrease the growth rate of multiple tumor cell types by activating protein kinase A (PKA) (16). The cAMP synthesis–activating agent forskolin (16) also inhibited BCR–Abl cells, whereas the inactive forskolin analog dideoxyforskolin had no effect (Fig. S2, *F*–*G*). Similar results were obtained with the PKA agonist 8Br-cAMP, but not with a cAMP analog that cannot activate PKA (17) (Fig. S2, *H*–*I*). These data demonstrate that drugs that increase cAMP levels selectively inhibit BCR–Abl-expressing cells.

Six uncharacterized compounds selectively inhibited BCR–Abl-expressing cells (DJ1, DJ2, DJ3, DJ12, DJ34, and DJ35; Fig. 2), but they did not directly target BCR–Abl (Fig. S3). Several of these compounds also inhibited the human CML cell lines MEG-01, KU-812, and K562, as well as the human Ph+ ALL cell line SD-1, which was not effectively killed by imatinib (Fig. S4), indicating that these compounds may serve as a starting point for development of alternative forms of therapy for both CML and Ph+ ALL patients.

### DJ34 selectively kills BCR–Abl-positive leukemia cells

**Figure 3:**
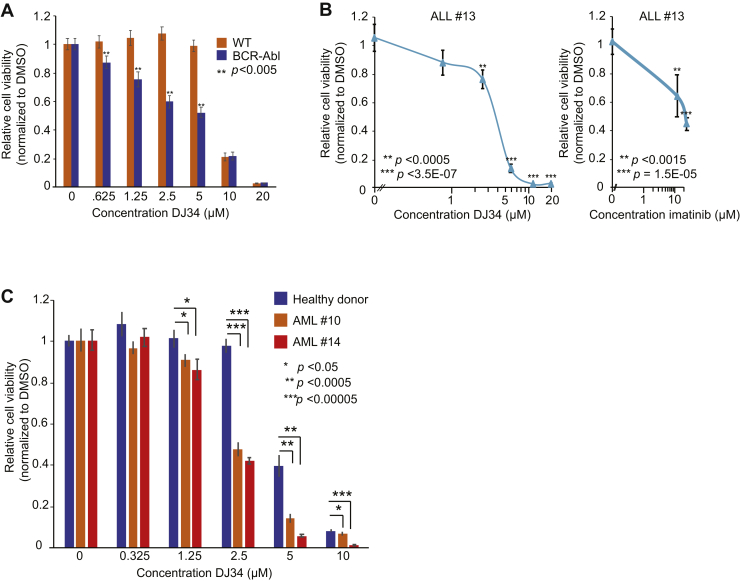
**DJ34 selectively targets BCR–Abl+ leukemia cells.***A*, BCR–Abl-transformed Ba/F3 cells are significantly more sensitive to DJ34 than isogenic control cells. Cells were incubated with increasing concentrations of DJ34 for 72 h, after which cell viability was analyzed by CellTiter-Glo. *B*, DJ34 efficiently kills primary Ph+ ALL cells. ALL cells were isolated from the bone marrow from a Ph+ ALL patient and incubated for 72 h with the indicated concentrations of DJ34 (*left*) or imatinib (*right*), after which cell viability was analyzed as in panel *A*. *C*, primary ALL cells are more sensitive to DJ34 than bone marrow cells derived from a healthy donor. Cells were isolated from bone marrow samples of an ALL patient (patient 10), a mixed B-ALL/AML patient (patient 14), and a healthy donor and incubated for 72 h with the indicated concentrations of DJ34, after which relative cell viability was analyzed by CellTiter-Glo. Error bars indicate standard deviation, and statistical significance was determined using Student's *t* test. ALL, acute lymphoid leukemia; AML, acute myelocytic leukemia; DMSO, dimethyl sulfoxide.

We analyzed the drug-like properties of the novel compounds using SwissADME (18) and found that DJ2, DJ12, and DJ35 have unfavorable drug-like properties, whereas DJ1 and DJ3 contain an alkyne group and a nitrile group, respectively, which can be unstable and chemically reactive *in vivo*. DJ34 was predicted to have excellent drug-like properties (Table S2, tab 1), which was confirmed by initial ADME (Absorption, Distribution, Metabolism, and Excretion)–pharmacokinetic (PK) analyses (Table S2, tab 2–6). We confirmed that freshly synthesized DJ34 targeted BCR–Abl-transformed Ba/F3 cells more efficiently than the isogenic parental cells (Fig. 3*A*). DJ34 also killed primary cancer cells derived from a Ph+ ALL patient more efficiently and at lower doses than imatinib (Fig. 3*B*). Importantly, blast cells derived from patients with ALL and mixed B-ALL/acute myelocytic leukemia (AML) were more sensitive to DJ34 than healthy bone marrow cells (Fig. 3*C*). Although analysis of a larger cohort of patients and healthy donors is required, these data suggest the existence of a potential therapeutic window.

### DJ34 inhibits the c-Myc transcriptional program and activates the p53 program

To better understand how DJ34 may inhibit cancer cells, we first performed phosphoflow cytometry experiments to investigate its effect on a panel of oncogenic signaling pathways. DJ34 had no effect on any of the examined phosphoproteins (Fig. S5*A*). Phosphorylation of S473 of AKT and Y694 of Signal Transducer and Activator of Transcription 5, both of which depend on BCR–Abl signaling, were also not affected by DJ34 (Fig. S5*B*), consistent with our observations that DJ34 does not directly inhibit BCR–Abl (Fig. S3). This shows that DJ34 does not affect several canonical oncogenic pathways.

**Figure 4:**
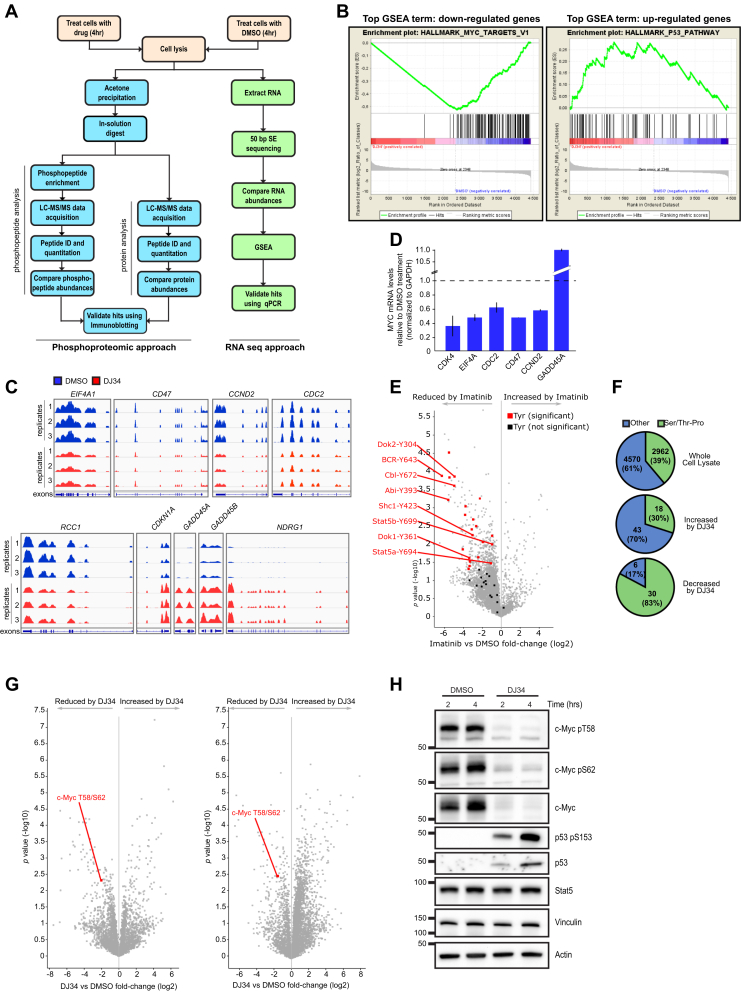
**Multiparameter analysis to determine the effect of DJ34 on transcription and cell signaling.***A*, workflow for the combined phosphoproteomic and RNA-Seq analysis of BCR–Abl-expressing cells treated with 20 μM DJ34. *B*, gene set enrichment analysis of significantly upregulated and downregulated transcripts following RNA-Seq, showing the top scoring enriched gene set terms. *C*, integrative genomics view of the mRNA levels of selected c-Myc target genes. *Y*-axes show RFPKM values. Range of the respective *y*-axes: *EIF4A1*: 0 to 1500; *CD47*: 0 to 1500; *CCND2*: 0 to 2000; *CDC2*: 0 to 1000; *RCC1*: 0 to 1000; *CDKN1A*: 0 to 4500; *GADD45A*: 0 to 200; *GADD45B*: 0 to 400; and *NDRG1*: 0 to 1000. *D*, RT–qPCR validation of selected c-Myc target genes after treatment with 20 μM DJ34. Error bars and standard deviation. *E*, volcano plot showing phosphosites modulated by imatinib. *F*, proportions of proline-directed phosphophorylation sites affected by DJ34. *G*, volcano plots of two biological repeats showing phosphosites regulated by DJ34 treatment. *H*, immunoblot analysis of BCR–Abl-expressing Ba/F3 cells treated with DMSO or 20 μM DJ34 for 2 and 4 h. DMSO, dimethyl sulfoxide; GSEA, gene set enrichment analysis; qPCR, quantitative PCR; SE, single end.

We then decided to apply a broader unbiased approach to determine how DJ34 may affect cells using a combination of RNA-Seq and MS-based phosphoproteomics for multiparameter analysis of the cellular programs altered by DJ34 (Fig. 4*A*). RNA-Seq analysis identified 1206 and 1705 gene transcripts that were at least twofold decreased or increased in abundance by DJ34 treatment, respectively (Table S3). Gene set enrichment analysis revealed that c-Myc–dependent genes were significantly enriched in the RNA-Seq data set as well as genes associated with the p53 pathway (Fig. 4*B*). Closer inspection confirmed that DJ34 downregulated genes, such as *EIF4*, *CD47*, *CDC2*, *CCND2*, and *RCC1*, which are activated by c-Myc (*e.g.*, (19); Fig. 4*C*). Conversely, treatment with DJ34 promoted the expression of genes that are inhibited by c-Myc, such as *NDRG1*, *CDKN1A*, and *GADD45* ((19, 20); Fig. 4*C*). Similar findings were obtained by RT–quantitative PCR (qPCR) analysis of several c-Myc target genes (Fig. 4*D*). Gene Ontology analysis of the genes upregulated by DJ34 revealed a significant enrichment of terms associated with reduced c-Myc function and/or increased p53 signaling, such as mitotic cell cycle, positive regulation of programmed cell death, and positive regulation of cell differentiation (Fig. S6*A*).

Phosphoproteomic analysis of cells treated with imatinib or DJ34 indicated that these compounds have distinct effects on cellular signaling pathways. We identified a similar number of unique phosphopeptides from dimethyl sulfoxide (DMSO)-, imatinib-, and DJ34-treated cells (Fig. S6*B*). Imatinib decreased the abundance of several tyrosine-phosphorylated peptides, including sites previously associated with BCR–Abl signaling (thus validating our approach; Fig. 4*E* and Table S4), whereas tyrosine phosphosites did not appear to be affected by DJ34 treatment (Table S5). Instead, a high proportion of the phosphosites reduced by DJ34 were proline-directed phosphosites (Fig. 4*F*), including the residues Thr58 and Ser62 on c-Myc (Fig. 4*G*), consistent with our RNA-Seq data indicating that DJ34 inhibits c-Myc activity (Fig. 4, *B*–*D*).

Validation of RNA-Seq and MS data by immunoblotting confirmed that not only phosphorylation of c-Myc Thr58 and Ser62 was decreased by DJ34 but also showed that total c-Myc protein levels were strongly reduced (Fig. 4*H*; total c-Myc levels were not detected by MS, most likely because it is a low-abundance protein that could only be detected after enrichment of phosphorylated peptides). Furthermore, p53 was undetectable in the lysates of DMSO-treated cells, whereas DJ34 treatment increased total p53 levels as well as Ser15-phosphorylated p53 (a marker of active p53; Fig. 4*H*). Together, these data indicate that DJ34 simultaneously inhibits c-Myc and activates p53.

### DJ34 induces depletion of c-Myc in a wide variety of tumor types

**Figure 5:**
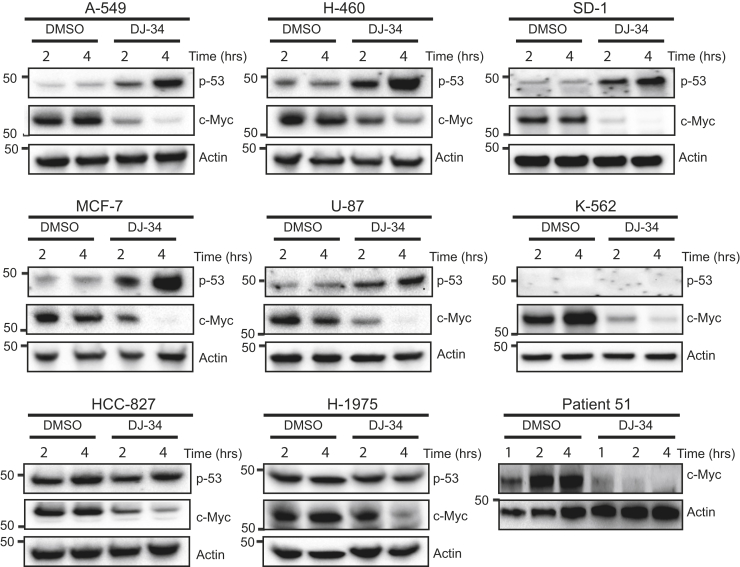
**DJ34 targets c-Myc and p53 in a variety of cancer cells.** Immunoblotting analysis for c-Myc, p53, and actin in various cancer cell lines treated with DMSO or 20 μM DJ34 for 2 and 4 h. In addition, bone marrow cells derived from an acute myelocytic leukemia patient were treated with DMSO or DJ34 for 1, 2, and 4 h, and c-Myc and actin levels were analyzed by immunoblotting (patient 51, *bottom right panel*). DMSO, dimethyl sulfoxide.

A compound that both inhibits c-Myc while stabilizing p53 could provide a valuable therapeutic agent not only for leukemia but also for a wide range of cancers (6, 21, 22, 23). We found that DJ34 treatment resulted in c-Myc depletion in all cancer cell lines tested, including glioblastoma, breast cancer, and lung cancer cell lines (Fig. 5). DJ34 treatment depleted c-Myc in primary human AML blasts as well (Fig. 5), showing that the anti-c-Myc effect of DJ34 is not restricted to laboratory cell lines. Furthermore, DJ34 treatment increased p53 levels for almost all cancer cell lines that exhibited low p53 levels under control conditions (Fig. 5). This included the lung cancer cell lines A-549 and H-460, the breast cancer cell line MCF-7, and the glioblastoma cell line U-87, all of which express WT p53 (24). The same was observed for the leukemia cell line SD-1 (unknown p53 status), whereas p53 was undetectable in the p53-mutant CML cell line K562 (25). Two other p53-mutant cell lines, H-1975 (R273H gain-of-function mutation) and HCC-827 (V218DEL) (26, 27), exhibited high basal p53 levels that were not increased by DJ34. Together, these data show that DJ34 treatment broadly inhibits c-Myc levels while at the same time activating p53.

### DJ34-induced cellular depletion of c-Myc requires an intact STIP1 homology and U-box containing protein 1/C-terminus of HSC70-interacting pathway

We wished to better understand the cellular requirements for the DJ34-induced reduction in c-Myc. The proteasomal inhibitor MG132 prevented DJ34-induced c-Myc depletion (Fig. S7*A*), suggesting that DJ34 treatment leads to proteasomal depletion of c-Myc. In many cancers, proteasomal degradation of c-Myc is prevented by increased phosphorylation of Ser62. This can occur *via* either hyperactive proline-directed kinases, including mitogen-activated protein kinases and cyclin-dependent kinases or inactivation of the phosphatase PP2A (28). DJ34 primarily downregulated proline-directed phosphorylation sites (Fig. 4*F*), so we assessed whether DJ34 induces proteasomal depletion of c-Myc *via* regulating its phosphorylation status. *In vitro* kinase analysis using the KINOME*scan* platform (www.discoverx.com) revealed that DJ34 had moderate or no effect on the activity of 55 kinases (Table S6), including the *bona fide* c-Myc kinases ERK and Cdk2. In addition, pretreatment with the potent PP2A inhibitor okadaic acid did not prevent DJ34-induced depletion of c-Myc (Fig. S7B; phosphorylation of the direct PP2A target Akt is shown as control (29)). These data indicate that DJ34 does not cause c-Myc depletion by targeting kinases or phosphatases known to regulate c-Myc proteasomal degradation.

We next tested the effect of DJ34 on other proteins known to regulate c-Myc proteasomal degradation. Certain Skp, Cullin, F-box (SCF) complexes can ubiquitinate c-Myc to target it for destruction (28). SCF activity requires prior neddylation of the Cullin RING ligase subunit by the ubiquitin-like protein Nedd8 (30). SCF activity can be efficiently inhibited by the small-molecule inhibitor MLN4924, which targets the E1 Nedd8-activating enzyme (31). While pretreatment of cells with MLN4924 considerably increased c-Myc levels, DJ34 treatment still resulted in depletion of a large proportion of c-Myc (Fig. S7*C*). Treatment with DJ34 also induced strong c-Myc depletion in *FBXW7*^−/−^ cells (Fig. S7*D*), which lack F-box and WD repeat domain-containing 7 (FBW7), which is a critical component of the main SCF responsible for c-Myc degradation (32). We did observe that the rate of c-Myc depletion was slower in *FBXW7*^*−/−*^ cells than in WT cells (see graph in Fig. S7*D*), suggesting that part of the c-Myc pool might be degraded by FBW7 on DJ34 treatment. Alternatively, it is possible that in *FBXW7*^*−/−*^ cells, the c-Myc degradation machinery is saturated because of the high levels of basal c-Myc, resulting in an apparent reduction in the rate of DJ34-induced c-Myc depletion. Consistent with the latter hypothesis, the levels of the FBW7 target cyclin E (33) were not affected by DJ34 (Fig. S7*E*), arguing against the possibility that DJ34 directly activates the SCF^FBW7^ complex to degrade c-Myc.

Next, we focused on CHIP (also known as STIP1 homology and U-Box containing protein 1), which is an E3 ubiquitin ligase that mediates c-Myc depletion independently of the phosphorylation status of c-Myc. Strikingly, even after 6 h of DJ34 treatment, CHIP^*−/−*^ mouse embryo fibroblasts still had not succeeded in degrading c-Myc (Fig. S7*F*), showing that an intact CHIP pathway is required for depletion of c-Myc by DJ34.

### DJ34 induces depletion of c-MYC mRNA

**Figure 6:**
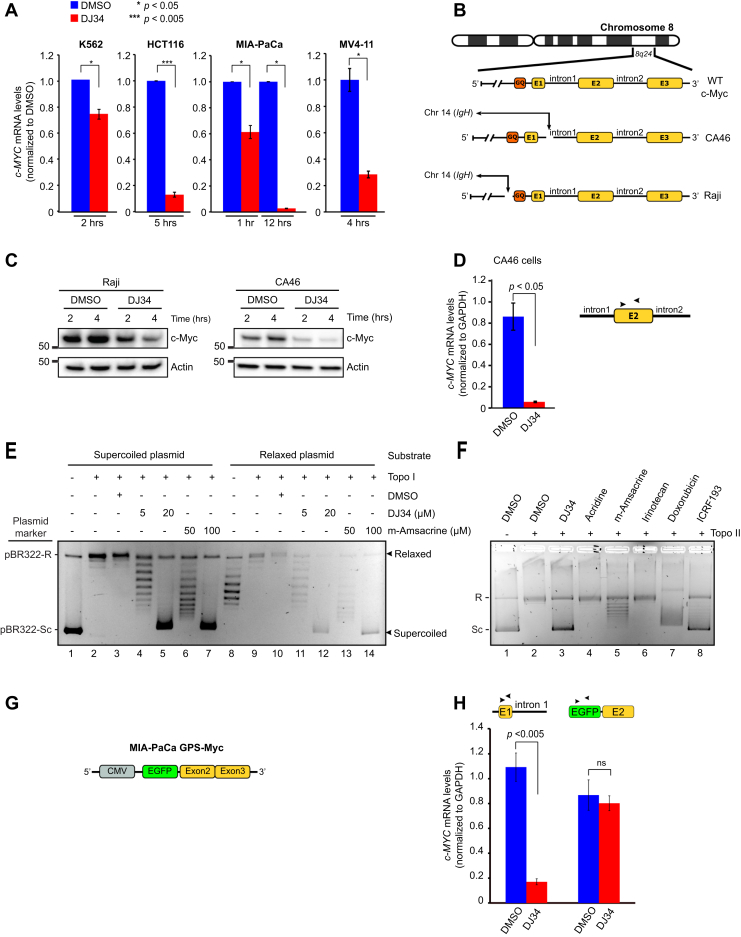
**DJ34 intercalates the DNA and inhibits topo II but not topo I.***A*, RT–qPCR analysis of *c-MYC* mRNA levels after K562, MV4-11, HCT116, and MIA–PaCa cells were treated with DMSO or 20 μM DJ34 for the indicated time points. mRNA levels were first normalized to GAPDH and then to the DMSO treatment. *B*, schematic overview of *c-MYC* status in nontranslocated (WT) cells compared with the lymphoma cell lines CA46 and Raji, both of which possess a reciprocal translocation between *IgH* and *c-MYC*. The translocation in CA46 cells leads to loss of the G4-quadruplex, whereas in Raji cells, the G4-quadruplex is retained. *C*, immunoblot analysis of Raji and CA46 cells treated with DMSO or 20 μM DJ34 for 2 and 4 h. *D*, RT–qPCR analysis of *c-MYC* mRNA levels in CA46 cells treated with DMSO or 20 μM DJ34 for 4 h. Primer pairs were designed to target exon 2 (as depicted above the bar graph), which almost exclusively measures levels of the translocated *c-MYC* allele, because expression of the nontranslocated allele is approximately 1000-fold lower than the translocated allele (36). *E*, DNA intercalation/topo I inhibition assay showing that DJ34 intercalates the DNA but does not inhibit topo I (see Fig. S7*I* for a detailed explanation of the assay). Supercoiled and relaxed plasmid substrates were incubated with different compounds and purified topo I as indicated, after which the products were analyzed by agarose gel electrophoresis. The plasmid marker provided in the assay kit is the 4361 bp plasmid pBR322 in either relaxed (R) or supercoiled (Sc) form. *F*, DJ34 inhibits topo II. Supercoiled plasmid substrate was incubated with topo II and various compounds as indicated above the figure, after which the products were analyzed by agarose gel electrophoresis. Same plasmid marker was used as in (*E*). All compounds were used at 10 μM, except DJ34 which was used at 20 μM. *G*, illustration depicting the *c-MYC* cDNA sequence randomly integrated into the genome of MIA PaCa cells (MIA–PaCa GPS–Myc). This cDNA lacks exon 1 and all introns. *H*, RT–qPCR analysis of *c-MYC* mRNA levels after MIA–PaCa GFP–Myc cells were treated with DMSO or 20 μM DJ34 for 4 h. Primers were designed targeting different regions of *c-MYC* (depicted above each set of bars) to distinguish between mRNA generated from WT *c-MYC* and cDNA-encoded *c-MYC*. CMV, cytomegalovirus; DMSO, dimethyl sulfoxide; EGFP, enhanced GFP; ns, not significant.

To further delineate the process of c-Myc depletion, we combined DJ34 with the ribosome inhibitor cycloheximide and monitored c-Myc protein levels over time. Interestingly, DJ34 did not appear to accelerate cycloheximide-induced depletion of c-Myc (Fig. S7*G*), suggesting that DJ34 primarily acts upstream of the ribosome. We used qPCR to determine whether DJ34 has a pretranslational effect on c-Myc, which revealed a significant reduction in *c-MYC* mRNA levels on DJ34 treatment in all cell lines tested, including K562, MV4-11, HCT-116, and MIA–PaCa cells (Fig. 6*A*). These data suggest that DJ34 inhibits transcription of *c-MYC*.

The reduction in c*-MYC* mRNA levels by DJ34 could be due to altered mRNA stability rather than inhibition of transcription. To distinguish between these possibilities, we blocked transcription with the strong RNA polymerase II inhibitor α-amanitin (34). We hypothesized that if DJ34 destabilizes mRNA, combining DJ34 with α-amanitin should result in more rapid loss of c-Myc, whereas there should be no added effect if DJ34 impedes *c-MYC* transcription, because mRNA synthesis is already fully blocked by α-amanitin. While treatment with α-amanitin resulted in efficient removal of c-Myc, α-amanitin did not appear to potentiate DJ34-induced loss of c-Myc (Fig. S7*H*), indicating that DJ34 and α-amanitin target the same process, *i.e.*, *c-MYC* transcription.

We reasoned that DJ34 might inhibit *c-MYC* transcription by interacting with regulatory elements in the *c-MYC* gene, such as the G-quadruplex in the promoter region (G4). This is a helical DNA structure formed by guanine tetrads, and several compounds can stabilize the quadruplex to inhibit *c-MYC* transcription (35). We examined c-Myc protein levels in two lymphoma cell lines in which genomic translocations of *IgH* with *c-MYC* have resulted in loss of the *c-MYC* promoter, either retaining or losing the G4-quadruplex (Raji and CA46, respectively; Fig. 6*B*) (36). DJ34 treatment downregulated c-Myc in both cell lines, suggesting that DJ34 does not require the G4-quadruplex to inhibit *c-MYC* transcription (Fig. 6*C*). RT–qPCR experiments with CA46 cells confirmed that DJ34 treatment resulted in a strong reduction in *c-MYC* mRNA (Fig. 6*D*). Together, these data show that DJ34 inhibits transcription of *c-MYC* and that this occurs independently of the G4-quadruplex.

### DJ34 intercalates DNA and inhibits topoisomerase II but not topoisomerase I

DJ34 contains three planar aromatic rings, a feature often found in DNA-intercalating compounds. Furthermore, compounds that inhibit topoisomerases through DNA intercalation have been shown to reduce *c-MYC* transcription (37,38). We therefore tested whether DJ34 intercalates and/or inhibits topoisomerase (topo) I/II to block *c-MYC* transcription. First, we performed a topo I DNA unwinding/intercalation assay, in which compounds are incubated with either a supercoiled or a relaxed substrate plasmid in the presence of topo I (see Fig. S7*I* for an overview). We found that incubation of DJ34 with topo I and either a supercoiled or a relaxed plasmid substrate exclusively produced supercoiled plasmids, showing that DJ34 does indeed intercalate but does not inhibit topo I (Fig. 6*E*; lanes 5 and 12). Notably, DJ34 intercalated at lower concentrations than m-amsacrine, a known DNA intercalator used to treat ALL (compare lanes 4–5 with lanes 6–7 and lanes 11–12 with lanes 13–14).

We then performed a topo II relaxation assay based on a supercoiled plasmid substrate that is relaxed by topo II, using m-amsacrine and doxorubicine (topo II poisons), 4,4'-(1,2-Dimethyl-1,2-ethanediyl)bis-2,6-piperazinedione (also known as ICRF-193, a topo II catalytic inhibitor), and irinotecan (topo I inhibitor with no effect on topo II) as controls. This revealed that DJ34 is a potent inhibitor of topo II, producing a strong supercoiled band on the DNA gel similar to that observed for 4,4'-(1,2-Dimethyl-1,2-ethanediyl)bis-2,6-piperazinedione (Fig. 6*F*; lanes 3 and 8, respectively). The DJ34 analog acridine did not inhibit topo II, suggesting specificity of DJ34, and determining the relevant molecular features of DJ34 is the focus of an ongoing follow-up study. Collectively, these data demonstrate that DJ34 is a DNA intercalator and a strong inhibitor of topo II, but not topo I.

It has previously been shown that intron 1 of the *c-MYC* gene contains a sequence that reduces *c-MYC* transcription because of torsional tension that arises during transcription, and the activity of topo II is essential for relieving this tension and maintaining high levels of *c-MYC* transcription; indeed, expression of plasmid-encoded *c-MYC* complementary DNA lacking this intron does not require topo II activity (37, 38, 39, 40). We analyzed c-Myc levels in the MIA–PaCa GPS–Myc cell line (41), which contains the endogenous WT *c-MYC* alleles in addition to a randomly integrated *dsRED–IRES–GFP–c-MYC* construct (Fig. 6*G*). This *c-MYC* complementary DNA lacks all introns including the aforementioned transcription-reducing intron 1, which is targeted by topo II to promote high *c-MYC* expression (37, 38, 39, 40). Interestingly, analysis of mRNA levels by RT–qPCR revealed that although endogenous *c-MYC* mRNA levels were strongly reduced, transcription of *GFP–c-MYC* was completely resistant to DJ34 (Fig. 6*H*). Taken together, although the precise mechanism remains to be unraveled, these data are consistent with a model in which DNA intercalation by DJ34 poisons topo II to interfere with *c-MYC* transcription.

### DJ34 induces cell cycle arrest, cell differentiation, and apoptosis

**Figure 7:**
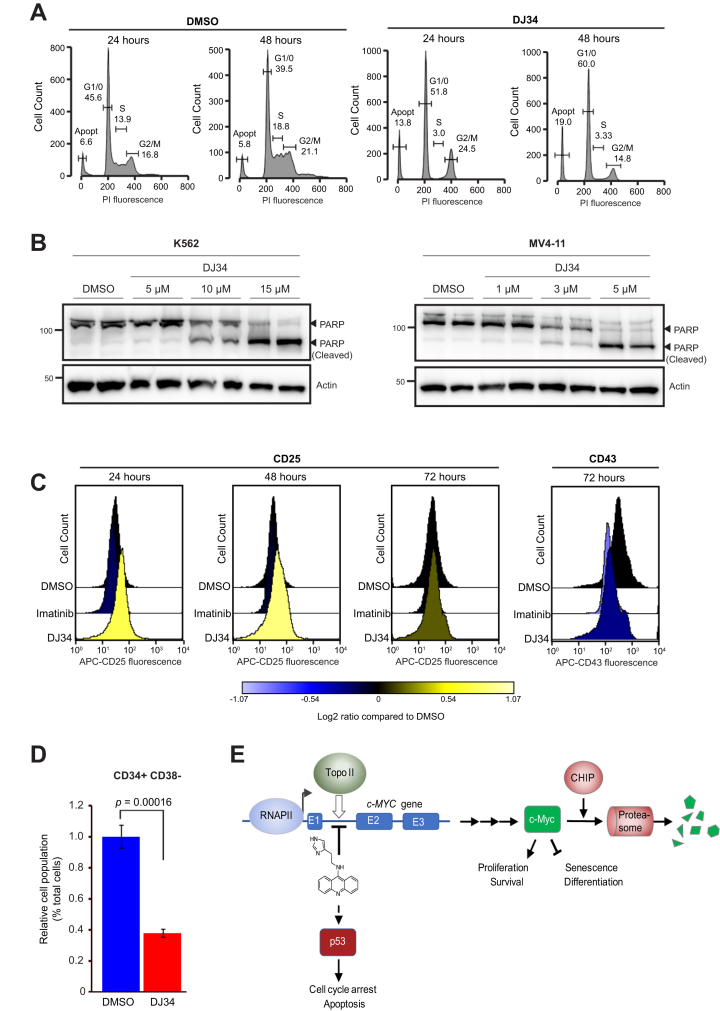
**DJ34 induces cell cycle arrest, apoptosis, and differentiation.***A*, cell cycle analysis of Ba/F3-BCR–Abl cells treated with DMSO or 20 μM DJ34 for 24 and 48 h. *B*, immunoblot analysis of K562 and MV4-11 cells treated with DMSO or 20 μM DJ34 at the indicated concentrations for 48 h using antibodies against PARP and actin. *C*, analysis by flow cytometry of the differentiation marker CD25 and the hematopoietic progenitor cell marker CD43 after treatment of Ba/F3-BCR–Abl cells with either 10 μM imatinib or 20 μM DJ34. About 10,000 cells were counted for each treatment and time point. *D*, CD34+CD38− LSCs are particularly sensitive to DJ34. Mononuclear cells were isolated from the bone marrow of a Ph+ CML patient and incubated for 24 h with 10 μM DJ34, after which the fraction of CD34+ CD38− cells in the total cell pool was analyzed by flow cytometry. Error bars and standard deviation. *E*, schematic overview of the findings of this study. DJ34 intercalates the DNA and inhibits topo II to activate p53 and to block transcription of *c-MYC*, which tentatively occurs in intron 1. c-Myc protein is then ubiquitinated and degraded by the proteasome through a pathway that requires CHIP. APC, anaphase promoting complex; DMSO, dimethyl sulfoxide; PARP, poly(ADP-ribose) polymerase-1.

We next investigated the physiological consequences of DJ34 treatment. Flow cytometry analysis showed that DJ34 treatment resulted in G1/G0 cell cycle arrest and apoptosis (Fig. 7*A*), which was confirmed by poly(ADP-ribose) polymerase cleavage in multiple cancer cell lines (Fig. 7*B* and Fig. S7*J*). In addition to causing apoptosis and cell cycle arrest, we found that DJ34 increased the expression levels of the differentiation marker CD25 while decreasing the levels of CD43, a marker of undifferentiated hematopoietic progenitors (Fig. 7*C*). DJ34 appeared to be more effective at inducing cell differentiation than imatinib, which is important, because c-Myc is known to mediate imatinib resistance by preventing imatinib-induced cell differentiation (42), and preventing differentiation is one of the mechanisms by which c-Myc promotes drug resistance in leukemia (43). Collectively, these data indicate that DJ34 triggers apoptosis, cell cycle arrest, and differentiation.

### DJ34 has anti-LSC activity

LSCs mediate disease relapse, and novel therapy that eradicates LSCs is expected to provide durable remission (44). It was previously shown that inhibiting c-Myc and reactivating p53 is an effective approach to eradicate LSCs (6). Given that DJ34 has dual c-Myc- and p53-targeting activity, we analyzed the effect of DJ34 on LSCs. We isolated bone marrow cells from a CML patient and treated the cells with either DMSO or with 10 μM DJ34. After 48 h, we analyzed the relative number of LSCs in the total cell population by flow cytometry. Importantly, DJ34 significantly reduced the relative number of CD34+ CD38− LSCs in the total pool of bone marrow–derived cancer cells (Fig. 7*D*), showing that LSCs are particularly sensitive to DJ34.

## Discussion

We performed a cell competition drug screen and identified several compounds that selectively inhibit BCR–Abl-expressing cells but not isogenic control cells. None of these compounds functioned as TKIs, suggesting they target parallel pathways that control proliferation and survival of transformed cells. Our findings support previous studies that showed that isogenic cell competition screens provide a powerful alternative approach to traditional cell viability–based drug screens, facilitating the identification of compounds that induce cell cycle arrest and cellular differentiation, as well as those that induce apoptosis. It has been argued that such phenotypic drug screens may help reduce the high attrition rates that have characterized drug development in the past decade (45, 46).

We identified a compound that may provide an effective lead structure for therapy. A model of our findings is shown in Figure 7*E*. DJ34 is a DNA intercalator and inhibits topo II, resulting in activation of p53 and inhibition of *c-MYC* transcription. As demonstrated by RNA-Seq, DJ34 did not appear to broadly inhibit transcription and showed relative selectivity for *c-MYC*, as the main genes affected by DJ34 were *c-MYC* itself and c-Myc target genes. The exact DNA sequence recognized by DJ34 remains to be identified, and exactly how DJ34 activates p53 remains to be established, but it is likely similar to activation of p53 by other topo II poisons.

DJ34-induced depletion of c-Myc was independent of the phosphorylation status of c-Myc. This is important because c-Myc–addicted tumors often stabilize c-Myc by interfering with its phosphorylation (47). Therefore, DJ34-based treatments may show clinical efficacy toward a broad range of c-Myc–addicted cancers, including cancers driven by oncogenic c-Myc mutants, such as the c-Myc-T58 and c-Myc-P57 mutations that are often found in Burkitt's lymphoma and which are refractory to phosphoregulation (48). Furthermore, DJ34 overcame MLN4924-dependent stabilization of c-Myc, suggesting that DJ34 induces c-Myc depletion independently of SCF ubiquitin ligases. This provides a therapeutic advantage because SCFs are often mutated and inactivated in cancer, including ALL (32, 49, 50). Indeed, loss of *FBXW7* has been shown to bolster leukemia-initiating stem cells in ALL by increasing c-Myc abundance, and inhibition of c-Myc resulted in remission in *fbxw7*-mutant mouse models of ALL (51). We believe that DJ34, or its derivatives, may therefore be particularly effective in these forms of ALL. We are currently testing the efficacy of DJ34 in mouse models, and our preliminary data indicate that DJ34 has a favorable ADME/Tox profile and is well tolerated by animals, all of which will be reported elsewhere.

c-Myc induces stemness and blocks cellular senescence and differentiation in many forms of cancer and promotes LSC survival in leukemia. Brief or even partial suppression of c-Myc can result in acute and sustained tumor regression, and dual targeting of p53 and c-Myc is a particularly effective strategy to kill LCSs (6). However, development of drugs that inhibit c-Myc has progressed slowly, primarily because c-Myc is widely considered undruggable. Given that DJ34 has potent anti-c-Myc activity in a broad range of cancers, that it can simultaneously activate p53, and that LSCs are particularly sensitive to DJ34, we believe it forms an excellent starting point for further development of anti-c-Myc cancer therapy.

## Experimental procedures

### Cell culture

Suspension cells (Ba/F3, K-562, MEG-01, MV4-11, SD-1, CA46, Raji, Daudi, 697, RS411, and KU-812) were cultured with RPMI-1640 with 10% fetal serum albumin and 1% penicillin/streptomycin. WT Ba/F3 cells also received 7.5 ng/ml IL3. Adherent cells (MIA PaCa, MIA–PaCa, GPS-Myc, A549, H460, MCF7, U87, HCC-827, H1975, SKBR3, MRC5, A375, MEWO, SK-MEL28, WM1366, HCT116, mouse embryo fibroblasts, and LN229) were cultured in Dulbecco's modified Eagle's medium with 10% fetal serum albumin and 1% penicillin/streptomycin. Primary patient cells were cultured with mononuclear cell basal medium (PromoCell, Heidelberg, Germany) supplemented with 10% SupplementMix (PromoCell, Heidelberg, Germany).

### Cell competition–based drug screen

Compounds were diluted in 10% DMSO and dispensed at the Chemical Biology Screening Platform of the Nordic Centre for Molecular Medicine (Oslo, Norway) into individual wells of a 384-well plate using an Echo acoustic liquid dispenser (Labcyte, San Jose, CA, USA) such that, after addition of cells, the compound concentration was 5 μM. Drug libraries that were used were the Sigma LOPAC1280 library and the ChemBioNet drug library (52).

A total of 1500 cells were mixed at a BCR–Abl:WT cell ratio of 1.3:1 in 50 μl cell culture medium (containing IL3). This ratio accounted for the slower growth rate of cells transfected with BCR–Abl and ensured that the ratio after 72 h treatment with DMSO was 1:1. In addition to library compounds, imatinib (5 μM) was used as a positive control in 16 wells of each 384-well plate. After 72 h, the number of GFP-positive (BCR–Abl) and red fluorescent protein–positive (WT) cells in each well was recorded by flow cytometry and used to calculate a cell ratio. The rationale for using 72 h was that during the course of our experiments we observed that in the presence of most drugs the cells had undergone at least several doublings after this amount of time (unperturbed Ba/F3 cells have a doubling time of approximately 12 h), which is essential for identifying a potential *in vitro* therapeutic index.

### Chemical synthesis

Synthesis of DJ34 and other compounds was performed by Hit2Lead, ChemBridge, Cambridge, UK. DJ34 is available on request.

### RNA-Seq

RNA-Seq was performed as previously described (53); see Supporting information section.

### RT–qPCR

RT–qPCR experiments were performed as previously described (54); see Supporting information section.

### *In vitro* kinase assays

Kinase assays were performed using the Omnia Kinase Assay according to the manufacturer's protocol (Life Technologies, Carlsbad, CA, USA); see Supporting information section.

### Proteomic and phosphoproteomic analyses

MS experiments were performed as previously described (55), see Supporting information section.

### Flow cytometry and phosphoflow cytometry

Flow cytometry was performed as previously described (56). See Supporting information section.

### Differentiation analysis

Bcr/Abl-expressing Ba/F3 cells were treated with DMSO, 10 μM imatinib, or 20 μM DJ34 for 24, 48, and 72 h. Cells were fixed as described previously (see cell cycle analysis), incubated with allophycocyanin-conjugated mouse anti-CD-25 and CD-43 antibodies (BD Biosciences, San Jose, CA, USA) and analyzed by flow cytometry for surface expression of CD25 and CD43.

### Apoptosis analysis

BCR–Abl-expressing Ba/F3 cells were treated with DMSO, 10 μM imatinib, and 20 μM DJ34 for 24, 48, and 72 h. Samples were then analyzed by flow cytometry, and cells undergoing apoptosis were quantified/gated according to changes in morphological and physical properties using forward and side-scattered light.

### Western blotting

Western blotting was performed as previously described (17); see Supporting information section.

### ADME–PK analysis

ADME–PK analysis was outsourced to the public CRO Cyprotex (www.cyprotex.com).

### DNA intercalation and topoisomerase assays

DNA intercalation and topoisomerase I and II activity assays were performed using a DNA Unwinding Assay Kit and a Human Topoisomerase II Relaxation Assay Kit, respectively, as described in the user manual (Inspiralis, Norwich, UK).

### Patient information

Patient 10—Caucasian male, ALL; patient 13—Caucasian male, Ph+ ALL; patient 14—Caucasian female, AML; patient 45—Caucasian male, CML.

## Data availability

All data are contained within the article, except RNA-Seq and MS data sets, which were deposited in the following public repositories: RNA-Seq data were deposited at Gene Expression Omnibus (GSE100678); MS data were deposited to the ProteomeXchange Consortium *via* Proteomics IDEntifications Database (PRIDE) repository (PXD018812). In addition, MS spectra were deposited to MS-Viewer (57). The search keys for these data sets are: ocnteamqeu (imatinib phosphoproteomic data); agzbem8ayy (imatinib proteomic data); p0kpemrznr and aeqwowtaj8 (DJ34 phosphoproteomic data repeats 1 and 2, respectively); and ijxqqn8lyw (DJ34 proteomic data).

## Ethical considerations

All samples were collected after obtaining written informed consent. The study was approved by the local ethical review board (REK2015/1012; Regional Ethics Committee South East, committee D) in accordance with the Declaration of Helsinki.

## Conflict of interest

J. M. E. has received research funding from ARIAD pharmaceuticals (now part of Takeda Oncology). The other authors do not declare conflict of interest.

## Supporting information

Figures S1–S12

JBC_JBC-2020-015285_11

JBC_JBC-2020-015285_12

JBC_JBC-2020-015285_13

JBC_JBC-2020-015285_14

JBC_JBC-2020-015285_15

JBC_JBC-2020-015285_16
