# Supplementary material for: A cell competition–based small molecule screen identifies a novel compound that induces dual c-Myc depletion and p53 activation
Source: J Biol Chem. 2020 Dec 17;296:100179. doi: 10.1074/jbc.RA120.015285 (PMC7948465; doi:10.1074/jbc.RA120.015285)

Supporting Information

"A cell competition-based drug screen identifies a novel compound that induces dual c-Myc depletion and p53 activation"

Dagim Shiferaw Tadele, Joseph Robertson, Richard Crispin, Maria C. Herrera, Marketa Chlubnova, Laure Piechaczyk, Pilar Ayuda-Durán, Sachin Kumar Singh, Tobias Gedde-Dahl, Yngvar Fløisand, Jørn Skavland, Jørgen Wesche, Bjørn-Tore Gjertsen and Jorrit M. Enserink

Material included:

- Supplemental Materials and Methods
- Description of Supporting Data not provided in pdf format
- Supplementary Figure Legends
- Supplementary Figures

Supplemental Materials and Methods

RNA sequencing

BCR-Abl-expressing Ba/F3 cells were treated with DMSO or 20 µM DJ34 in triplicate for 4 hrs. Total RNA was extracted using the RNeasy mini kit according to the manufacturer's protocol (Qiagen, Manchester, UK). Purity of the RNA was confirmed by bioanalyzer, and RNA transcriptome library construction and Illumina Hiseq 4000 was performed at BGI Tech solutions (Hong Kong). RNA sequencing (RNA Seq) analysis was performed by Hemispherian RS (Oslo, Norway).

Prior to follow-up analyses, a value of 0.1 was added to the mean FPKM RNA Seq readings of all significant genes, so that genes with a zero value in one condition were still assigned a fold-change value when DMSO and DJ34 were compared. Genes with FPKM RNA Seq readings lower than one in both conditions were removed. Integrative genomics viewer (IGV) plots showing RNA Seq data for specific genes were generated using the IGV visualization tool as described by Robinson et al(1). Gene set enrichment analysis (GSEA) was performed with GSEA software (2) using all genes that were significantly altered by DJ34 treatment. Metascape analysis (http://metascape.org (3)) was performed using only genes that were more than 2-fold up-regulated by DJ34 treatment. Enrichment cut-offs for Metascape were p-value<0.01, minimum gene count 3 and enrichment factor >1.5.

RT-qPCR

BCR-Abl expressing Ba/F3 cells were treated with DMSO or 20µM DJ34 for 4 hrs and total RNA were extracted as described for RNA sequencing. For each treatment, 100 ng of total RNA was used to synthesize cDNA using the QuantiTect reverse transcription kit according to the manufacturers protocol (Qiagen). RT-qPCR was performed using 2x fast syber green master mix (Foster City CA, USA), 0.2 µM of forward and reverse primers for the MYC target genes CDK4, EIf4e, and GAD45. Primers used for qPCR were 5'TGTTTGAGCATATAGACCAG3' forward and 5'AATCCCAACAACTTCTATTG3' reverse for CDK4, 5'CTACTGATGGACACTTCTAC3'

forward and 5’ACTTAGAGATCAATCGAAGG3’ reverse for EIF4E, 5’GAGAACGACATCAACATC3’ forward and 5’CTCGGACAAGGTCCA3’ reverse for GAD45, 5’TCGGGTAGTGGAAAACCAGC3’ forward and 5’TTCCTGTTGGTGAAGCTAACGTT3’ reverse for MYC (exon1), 5’Ccgaccagctggag3’ forward and 5’CAgcttctctgagacgagct3’ reverse for MYC (exon2), 5’CAGCACGACTTCTTCAAGTCCG3’ forward and 5’GTAGTTGTACTCCAGCTTGTGCC3’ reverse primers for MYC (EGFP-exon2), and 5’TCGTCCCGTAGACAAAATGGT3’ forward and 5’CGCCCAATACGGCCAAA3’ reverse for GAPDH.

Proteomics

BCR-Abl-expressing Ba/F3 cells were treated with DMSO, 10 μM imatinib or 20 μM DJ34 for 4 hrs. Cells were lysed using a Triton-based lysis buffer (150 mM NaCl, 25 mM Tris pH 7.4, 1% (w/v) Titon-X-100) containing a protease inhibitor cocktail (Calbiochem Set 1; Merck Millipore) and a phosphatase inhibitor cocktail (Thermo Fisher Scientific). Samples equating to 1 mg of protein were reduced using dithiotheitol (DTT), precipitated using -20°C acetone and proteolytically digested using 20 μg trypsin (Promega, Madison, WI, USA).

For the proteomic element of the workflow, approximately 50 μg of peptides was de-salted using C_{18} StageTips and analyzed by mass spectrometry (MS) to identify protein components.

The remaining peptides (approximately 950 μg) were processed for identification of phosphopeptides. Samples were de-salted using Oasis HLB sample extraction columns (Waters, Manchester, UK) and enriched for phosphopeptides using titanium dioxide enrichment beads (see Robertson et al (4) for details). Enriched fractions were de-salted twice using reverse-phase ZipTips containing C_{18} media (Merck Millipore), and analyzed by MS to identify phosphopeptides.

Tandem MS analysis (LC-MS/MS) of all samples was performed in triplicate using an Easy nLC1000 liquid chromatography (LC) system (Thermo Electron, Bremen, Germany) coupled to a QExactive Plus Hybrid Quadrupole-Orbitrap mass spectrometer (Thermo Electron) with a nanoelectrospray ion

source (EasySpray, Thermo Electron). The LC separation of peptides was performed using an EasySpray C18 analytical column (2 µm particle size, 100 Å, 75 µm inner diameter; Thermo Fisher Scientific). Peptides were separated over a 120 min solvent gradient from 2% to 30% (v/v) ACN in 0.1% (v/v) FA, after which the column was washed using 90% (v/v) ACN in 0.1% (v/v) FA for 20 min (flow rate 0.3 µL/min). All LC-MS/MS analyses were operated in data-dependent mode where the most intense peptides were automatically selected for fragmentation by high energy collision-induced dissociation.

Raw files from MS analyses were submitted to MaxQuant software (5) for peptide/protein identification using the Uniprot mouse database. MaxQuant output files (proteinGroups.txt for proteomic data and STY(sites).txt for phosphoproteomic data) were loaded into the Perseus software (6). Identifications from potential contaminants and reversed sequences were removed and intensities were transformed to log2. Identified phosphorylation sites were filtered only for those that were confidently localized (class I, localization probability ≥ 0.75). All zero intensity values were replaced using noise values of the normal distribution of each sample. For proteomic data, protein abundances were compared using LFQ intensity values and a two-sample Student's T-test (permutation-based FDR correction (250 randomizations), FDR cut-off: 0.05, S0: 0.1). For phosphoproteomic data, phosphosite abundances were compared using intensity values and a two-sample Student's T-test (p value cut off: 0.05). For display of phosphoproteomic data, volcano plots were generated in Perseus, with phosphosites labelled as significantly decreased according to the Student's T-test. Lists of all identified proteins and phosphosites, as well as those that were significantly affected by DJ34 or imatinib treatment, can be found in Supplementary Tables S4 and S5.

All proteomic/phosphoproteomic analyses involved one biological replicate and three technical replicates (i.e. three LC-MS/MS injections per sample), with the exception of DJ34 phosphoproteomic analysis in which two biological replicates were performed (each involving three technical replicates). Phosphorylation sites were deemed significant only if they were significantly up- or down-regulated in both biological repeat datasets.

Flow Cytometry and phosphoflow cytometry

BCR-Abl-expressing Ba/F3 cells were treated with DMSO or 20 µM DJ34 for 24 and 48 hours. Cells were washed in cold PBS and fixed in cold 70% (v/v) ethanol for 2 hours. Subsequently, fixed cells were stained with a solution containing 0.1% (w/v) Triton-X-100, 10 µg/ml propidium iodide and 100 µg/ml RNase A in PBS at 37°C for 10 min and analyzed by flow cytometry. For LSC analysis, primary patient cells were recovered by over-night culturing, then cells were treated with DMSO or 10 µM DJ 34 for 24 hours. Cells were washed in cold PBS and blocked with 0.5% BSA for 45 minutes. Cells were then stained with FITC conjugated anti-CD38 and PE conjugated anti-CD34 antibodies (Abcam, Cambridge, UK) for 45 minutes and analyzed by flow cytometry.

In vitro kinase assays

10 µg/ml of purified GST-Abl kinase domain was incubated with 10 µM of the Omnia Tyrosine Peptide Y6, 0.2 mM DTT, 10 µM of the compounds of interest, and 50 µM ATP. Fluorescence intensity was measured using the Envision 2104 Multilabel Reader (Perkin Elmer; Waltham, MA, USA). Fluorescence intensities were normalized to the first time-point measured.

Western blotting

Cells were treated with compounds or, as a control, DMSO. Unless stated otherwise, DJ34 was used at 20 µM and imatinib was used at 10 µM, and the incubation time was 4 hours. Cells were washed with PBS and lysed using Laemmli buffer or RIPA buffer (150 mM NaCl, 50 mM Tris-Hcl pH 8.0, 1% (w/v) Triton-X-100, 0.5% (w/v) sodium deoxycholate, 0.1% (w/v) SDS) containing a protease inhibitor cocktail (Calbiochem Set 1; Merck Millipore) and a phosphatase inhibitor cocktail (Thermo Fisher Scientific, Waltham, MA, USA). Lysates were centrifuged to remove insoluble material (22,000 *g*, 15 min, 4°C). Proteins were separated by SDS-PAGE and transferred to a nitrocellulose membrane (Bio-Rad, Hercules, CA, USA). Membranes were blocked using blocking buffer (5%

(w/v) skim milk (Sigma-Aldrich) in TBS containing 0.1% (v/v) Tween-20 (TBS-T)) and probed overnight at 4°C with primary antibodies incubated in blocking buffer. Membranes were washed for 30 mins using TBS-T, and then incubated with the appropriate horseradish peroxidase (HRP)-conjugated secondary antibodies (GE Healthcare, USA) in blocking buffer for 60 min. Membranes were washed for 30 min using TBS, incubated with enhanced chemiluminiscent substrate for HRP (Thermo Fisher Scientific) for 5 min and scanned for visualization. For a list of antibodies see Supplemental Table S1.

Plasmids

Commercially available plasmids used were pcDNA3::p210-BCR-Abl, which was a gift from Warren Pear (Addgene plasmid # 27481, Cambridge MA, USA); GFP expression, pRNAT-H1.1/Hygro plasmid from Genscript (Piscataway NJ, USA); RFP expression, pmCherry-N1 from Clontech (Mountain View CA, USA). The plasmid used for expression of recombinant Abl kinase domain (c-Abl amino acids 220-498) for in vitro kinase assays was a kind gift from Michael W. Deininger (University of Utah Huntsman Cancer Institute, UT, USA). All transfections were performed using the Amaxa Biosystems Nucleofector II (Lonza, Cologne, Germany).

Description of Supporting Data not provided in pdf format:

- **Table S1:** Excel file containing a list of antibodies used in this study

- **Table S2:** Excel file containing information regarding the drug likeness of DJ34

- **Table S3:** Excel file containing RNAseq data

- **Table S4:** Excel file containing MS data of imatinib-treated cells

- **Table S5:** Excel file containing MS data of DJ34-treated cells

- **Table S6:** Excel file containing KinomeScan data

Supplementary Figure Legends

Supplementary Figure S1. (**A**) Overview of the name, known molecular targets and structure of the compounds that increase the relative competitiveness of BCR-Abl-expressing cells relative to isogenic control cells. The results obtained with the competition assay and cell viability assay are also shown. All values were normalized to DMSO. (**B**) Effect of the JAK2 inhibitors Ruxolitinib, S-Ruxolitinib and Tofacitinib on the viability of WT and BCR-Abl-expressing Ba/F3 cells. Cells were treated with the indicated doses of drugs for 72 hrs after which cell viability was assessed by CellTiter-Glo. (**C**) Relative viability of BCR-Abl-expressing or WT cells treated with the JAK2 kinase inhibitor AZD1480 combined with either DMSO or imatinib. Error bars: standard deviation.

Supplementary Figure S1

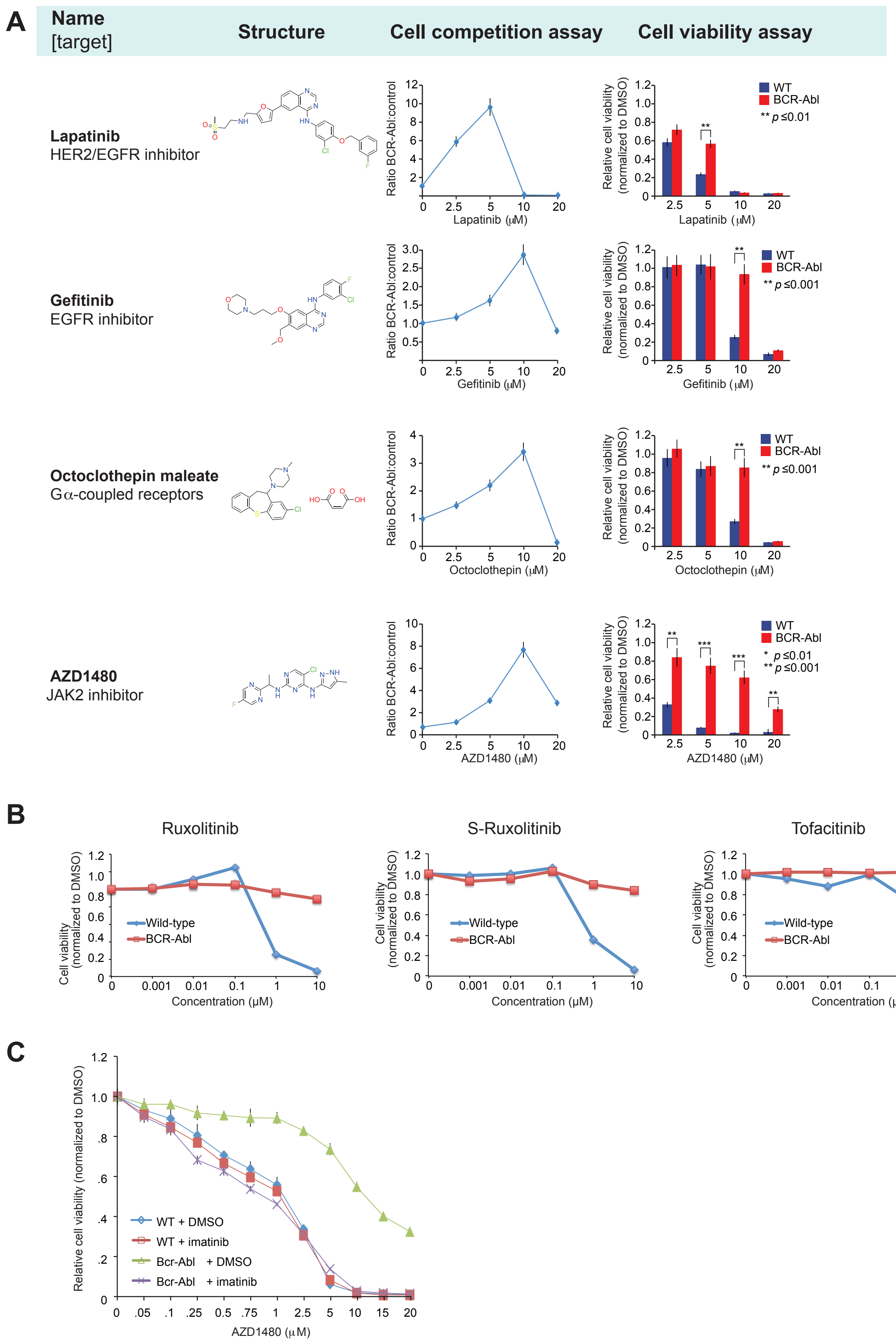

Supplementary Figure S2. (**A**) The ratio of BCR-Abl-expressing cells over WT Ba/F3 cells following treatment for 72 hours with the topoisomerase II inhibitors ICRF-193 (upper x axis) and Etoposide, Doxorubicin and Camptothecin (lower x axis). Ratios were calculated using the cell competition-based assay (see Fig. 1). (**B-G**) Cell viability of BCR-Abl-expressing or WT Ba/F3 cells following treatment with the PDE inhibitors IBMX (*B*), Ibidulast (*C*), Rolipram (*D*) and Zardaverine (*E*); the adenylate cyclase activator forskolin (*F*), and the inactive forskolin analogue dideoxyforskolin for 72 hours (*G*). (**H, I**) Effect of the cAMP analogues 8Br-cAMP or 007 on the viability and competitiveness of BCR-Abl-expressing and WT Ba/F3 cells after 72 hours treatment.

Supplementary Figure S2

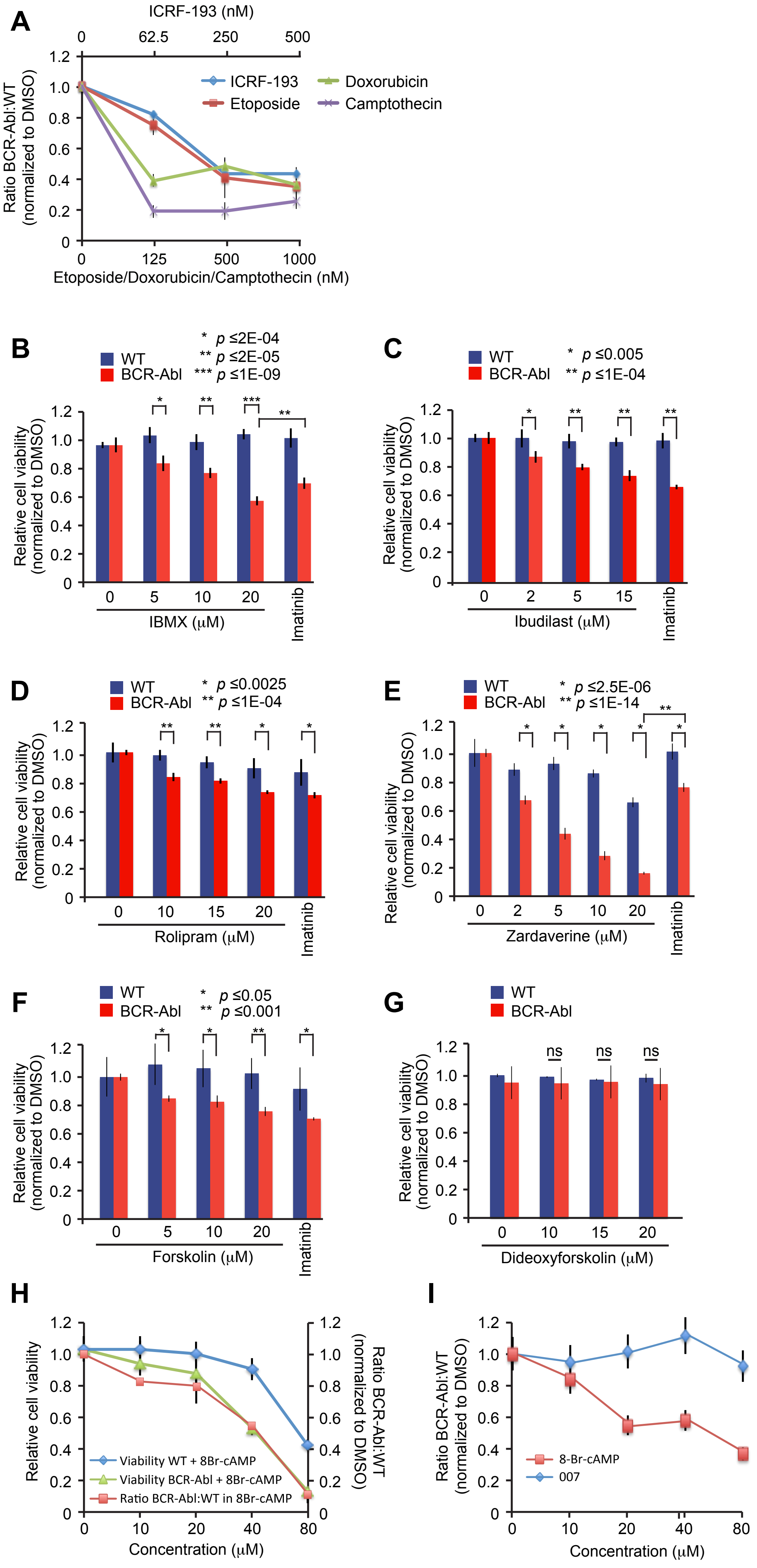

Supplementary Figure S3. Relative kinase activity of purified recombinant Abl kinase domain following treatment with DJ1, DJ2, DJ3, DJ34 and DJ35. DMSO treatment was used as a negative control, and the Abl kinase inhibitor ponatinib was used as a positive control. All data were normalized to timepoint 0.

Supplementary Figure S3

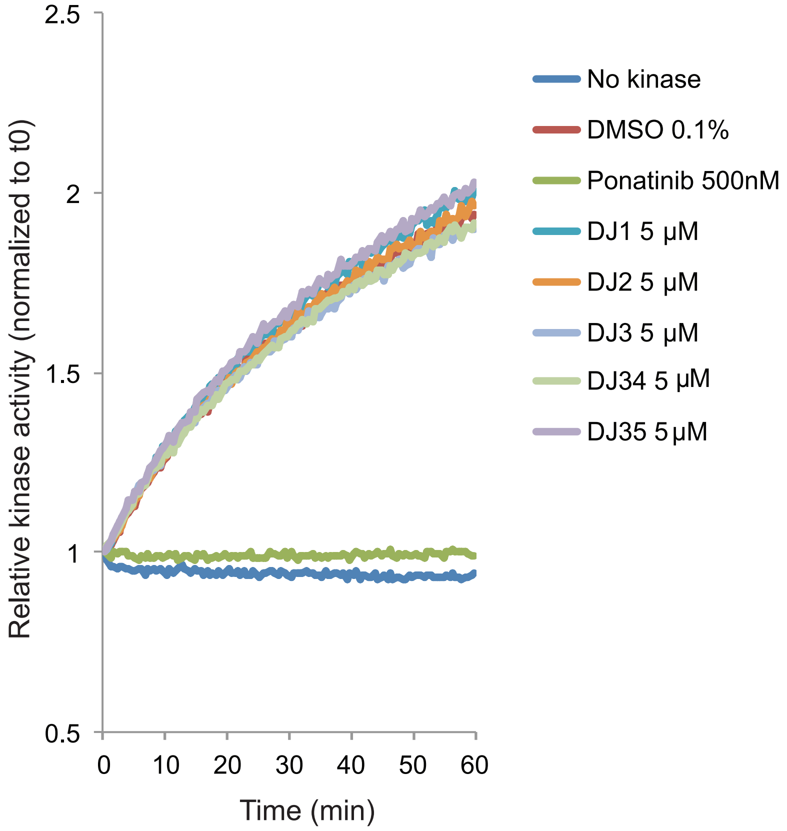

Supplementary Figure S4. (A-D) Cell viability of human leukemia cell lines following 72 hours treatment with DJ1, DJ2, DJ3, DJ12, DJ34, DJ35, 8Br-cAMP or forskolin. Cell lines used were the CML cell lines MEG-01 (*A*), KU-812 (*B*) and K562 (*C*), and SD-1 (Ph+ ALL) (*D*).

Supplementary Figure S4

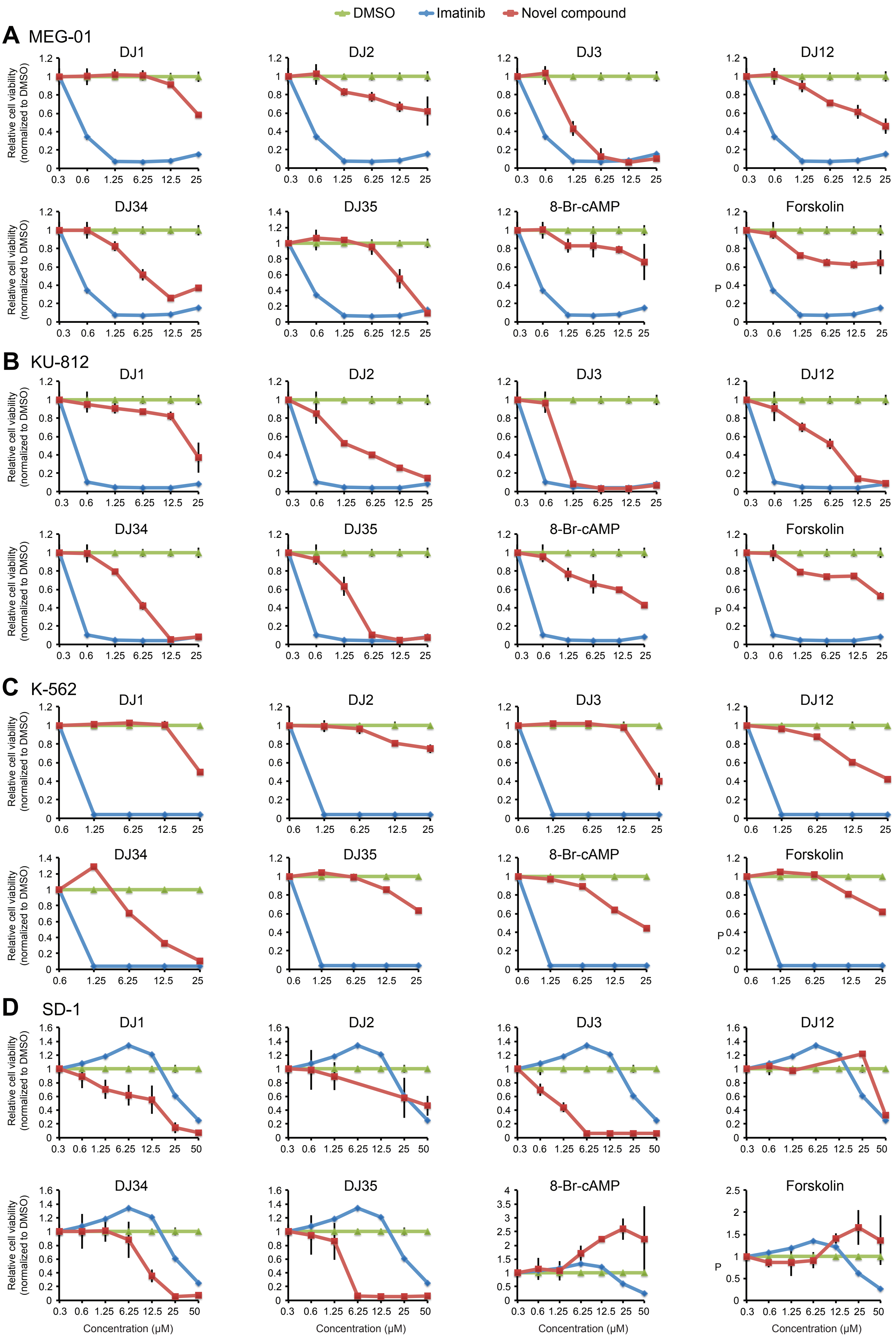

Supplementary Figure S5. (A) Phospho-flow cytometry analysis showing the effect of 20 μM DJ34 or 10 μM imatinib on the levels of phosphorylation sites known to be components of oncogenic signaling pathways. (**B**) Immunoblot analysis of BCR-Abl-expressing Ba/F3 cells treated with 20 μM DJ34 or 10 μM imatinib for 4 hrs. Membranes were probed with antibodies targeting phosphorylation sites that are known components of signaling pathways downstream of BCR-Abl.

Supplementary Figure S5

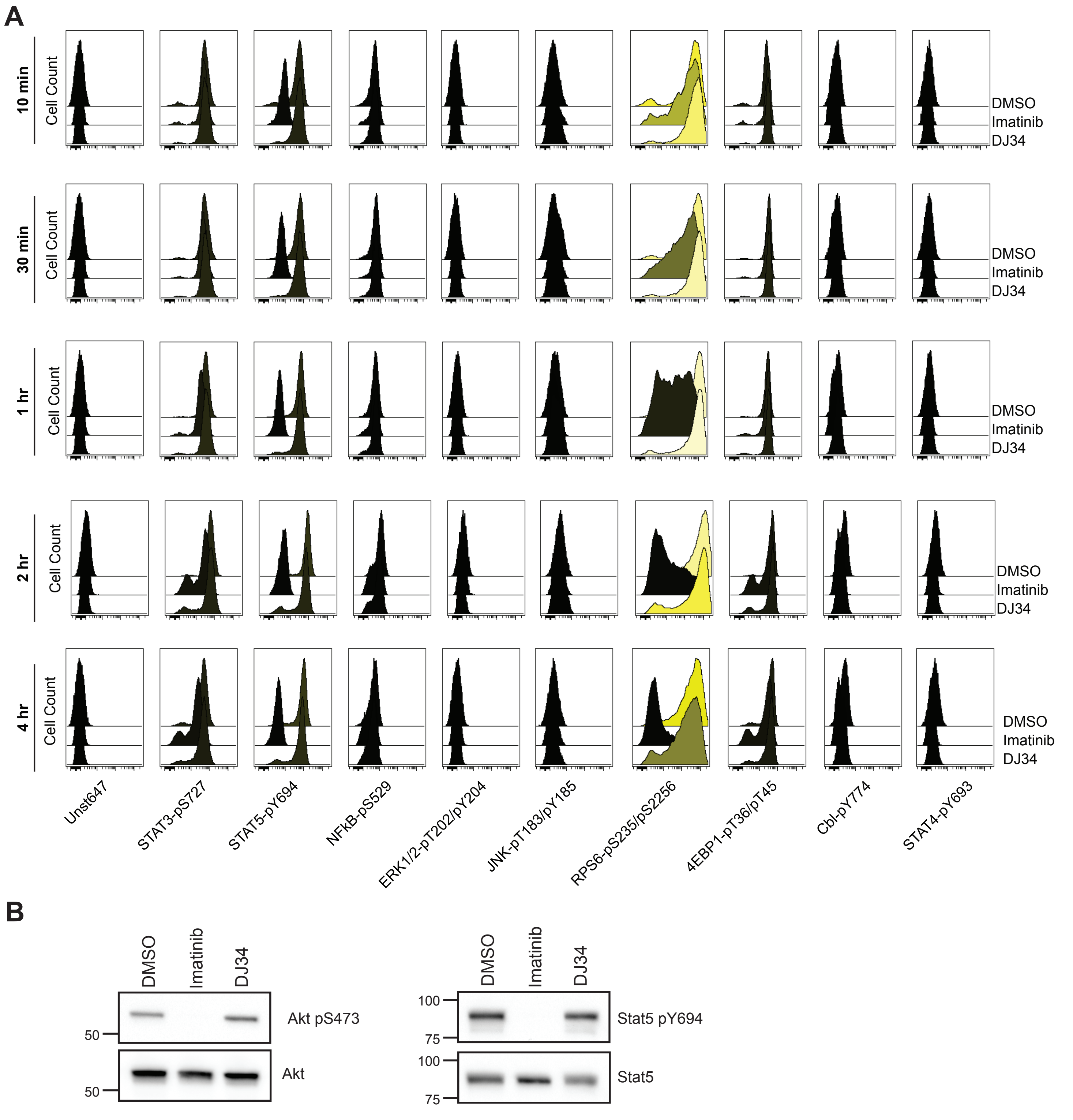

Supplementary Figure S6. (**A**) Metascape analysis of the 1705 transcripts that increased at least 2-fold in abundance following RNA-seq analysis of DJ34-treated cells. (**B**) Total numbers of unique phosphopeptides identified by MS from cells treated with 10 μM imatinib or 20 μM DJ34.

Supplementary Figure S6

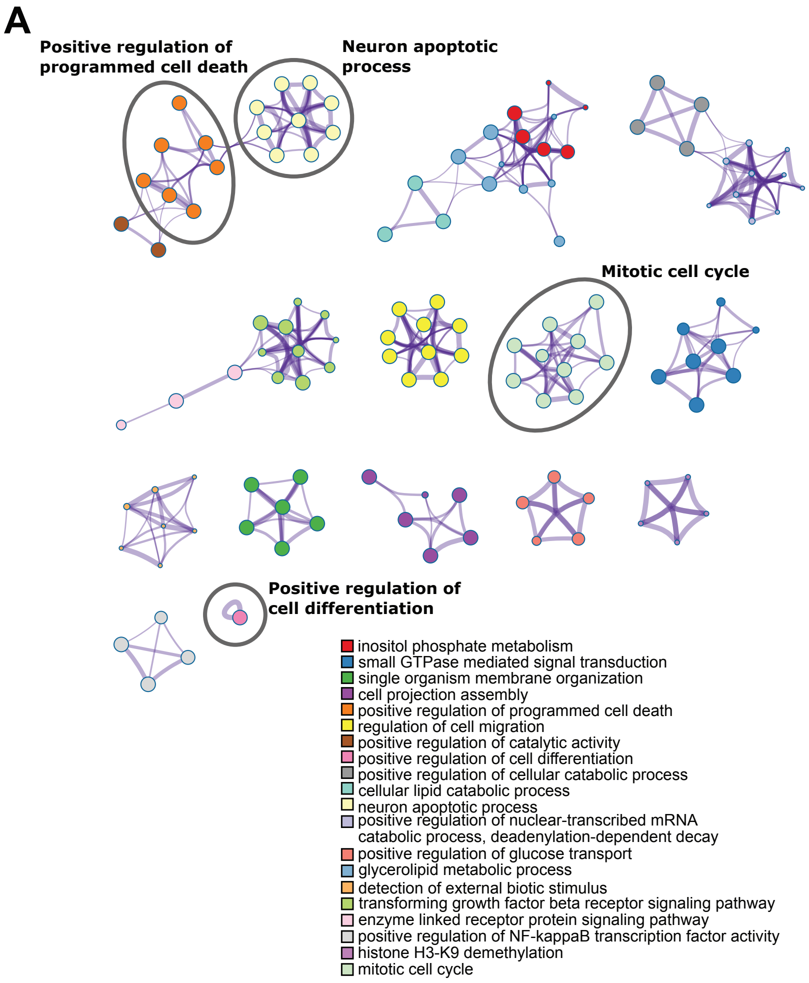

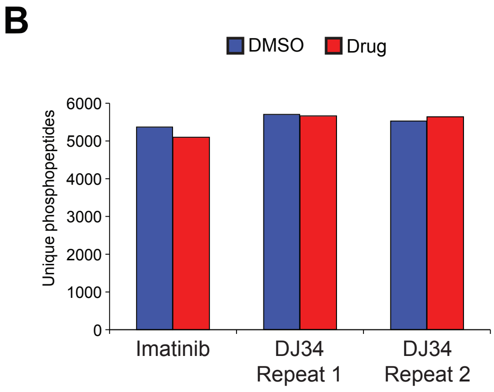

Supplementary Figure S7. **(A)** Immunoblot analysis of BCR-Abl-expressing Ba/F3 cells treated with 20 μM DJ34 for 1, 2 or 4hrs after pre-treatment for 1hr with 10 μM MG132. (**B** and **C**) Immunoblot analyses of BCR-Abl-expressing Ba/F3 cells treated with DJ34 after 1 hr pre-treatment with either 500 nM okadaic acid (OA; *B*) or 10 μM MLN4924 (*C*). (**D**) DJ34-induced c-Myc depletion does not require SCF^{FBW7}. WT HCT116 cells or isogenic $FBXW7^{-/-}$ knock-out cells were mock-treated or treated with 20 μM DJ34 for the indicated times, after which cell lysates were analyzed by immunoblotting with antibodies against c-Myc and Actin. Quantification of c-Myc bands across three technical repeat experiments is displayed below the immunoblotting images. (**E**) Immunoblot analysis showing the effect of 20 μM DJ34 on cyclin E levels in three different cancer cell lines. (**F**) DJ34-induced c-Myc depletion is mediated by CHIP. WT MEFs or CHIP-/- MEFs were treated and analyzed as in (*D*). (**G**) DJ34 down-regulates c-Myc upstream of the ribosome. Cells were pretreated with 10 μg/ml cycloheximide for 10 mins, followed by treatment with DMSO or DJ34 for the indicated times, after which cell lysates were analyzed by immunoblotting with c-Myc and Actin antibodies. For comparison, cells were also treated with DJ34 alone (i.e. 10 mins DMSO pre-treatment followed by DJ34). (**H**) DJ34 does not accelerate alpha-amanitin-induced loss of c-Myc. Cells were treated with 50 μM alpha-amanitin and 20 μM DJ34 as indicated, after which cell lysates were analyzed by western blotting with c-Myc and Actin antibodies. (**I**) Schematic overview of the topo I DNA unwinding/intercalation assay (with both supercoiled and relaxed DNA plasmids) outlining the possible results for a compound with different DNA intercalating and topo I inhibitory properties. Lanes represent the different properties in relation to whether or not a compound can intercalate DNA or inhibit topo I, respectively, which is further summarized in the table underneath the two panels. In brief, in this hypothetical experiment a compound that neither intercalates nor inhibits topo I will produce a relaxed plasmid for both substrates (compound A, lane 3 in both panels), whereas a compound that does intercalate but does not inhibit topo I will produce supercoiled plasmids, regardless of the substrate used (compound B, lane 4 in both panels). Finally, a compound that both intercalates DNA and inhibits topo I will produce a supercoiled plasmid from a supercoiled substrate, and a relaxed plasmid from a relaxed substrate (compound C, lane 5 in both panels). (**J**) Immunoblot analysis of RS4-11 and LN229 cells treated with DMSO or DJ34 at the indicated concentrations for 48 hrs. Cell lysates were analyzed with antibodies against PARP and Actin.

Supplementary Figure S7

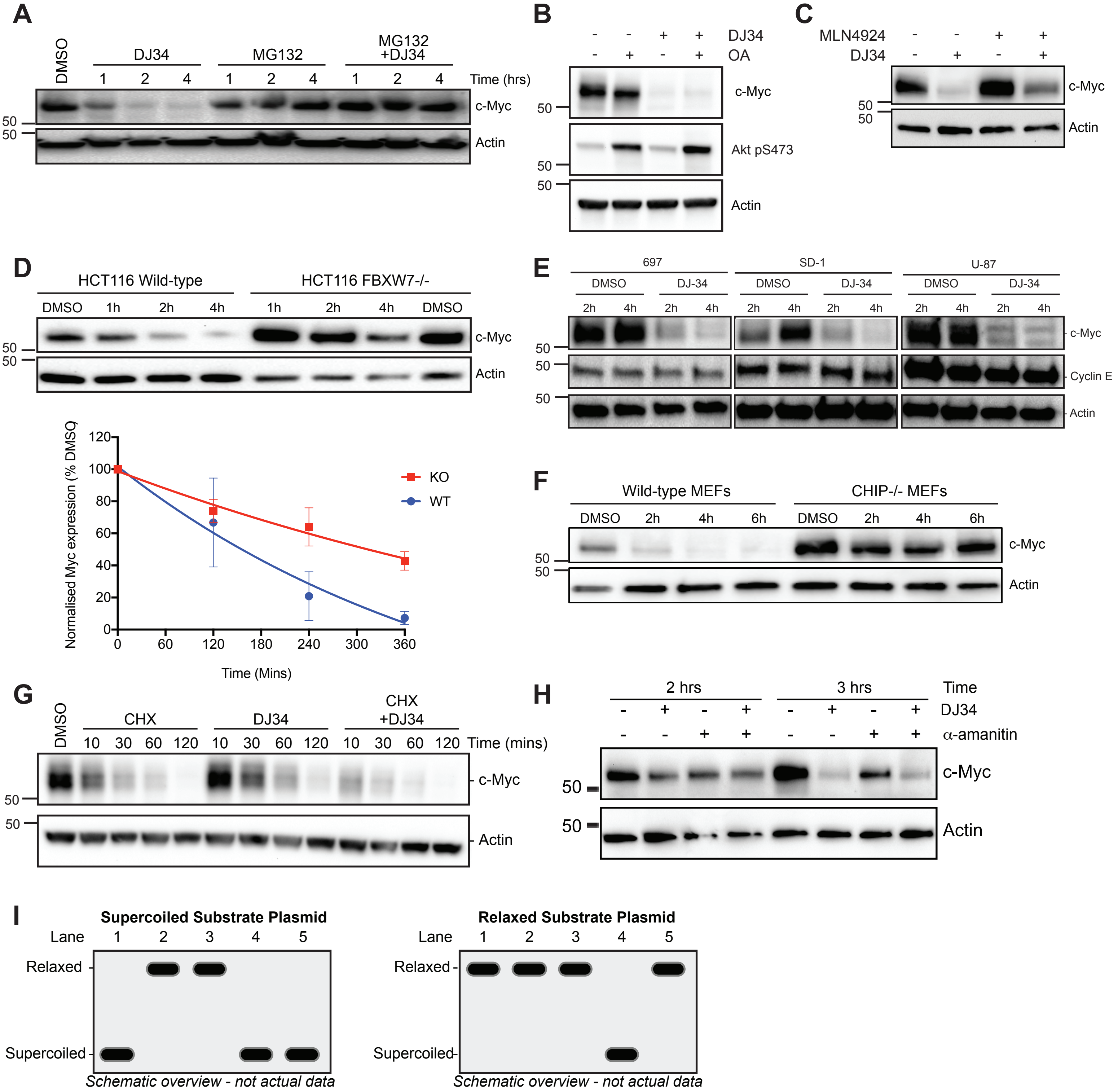

	Present in the assay		Effect of the compound		
**Lane**	**Compound**	**Topo I**	**Intercalation**	**Topo I inhibition**	**Effect on supercoiled (S-plasmid) and Relaxed (R-plasmid) substrate**
1	DMSO (control)	-	N/A	N/A	Substrate-only negative control: No effect
2	DMSO (control)	+	N/A	N/A	Topo I positive control: S-plasmid becomes relaxed, R-plasmid remains relaxed
3	Compound A	+	-	-	No effect of the compound: S-plasmid becomes relaxed, R-plasmid remains relaxed
4	Compound B	+	+	-	S-plasmid remains supercoiled and R-plasmid also becomes supercoiled
5	Compound C	+	+	+	S-plasmid remains supercoiled and R-plasmid remains relaxed

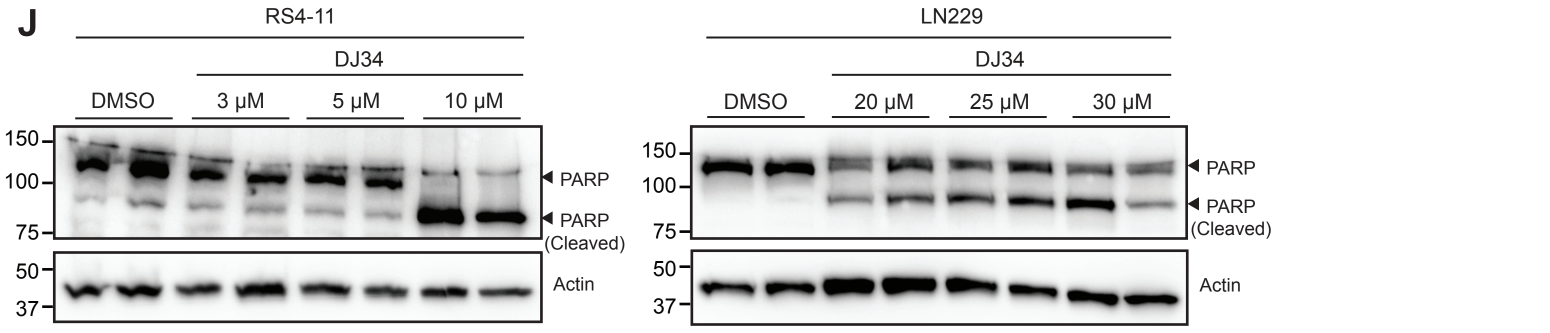

Supplemental Figure S8. Uncropped western blots shown in Figure 4.

Supplemental Figure S8

Uncropped Westerns Shown in Figure 4

Fig 4H: Myc pS62

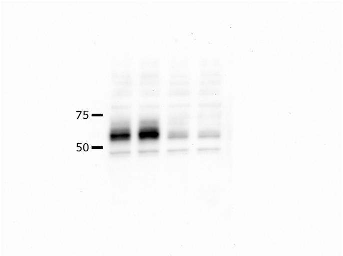

Fig 4H: Myc pT58

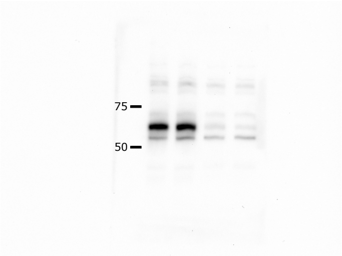

Fig 4H: Myc

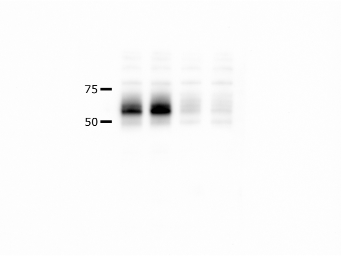

Fig 4H: p53 pS15

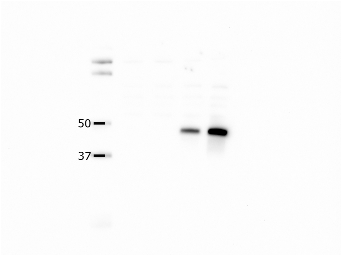

Fig 4H: p53

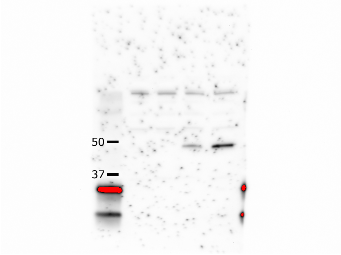

Fig 4H: Stat5

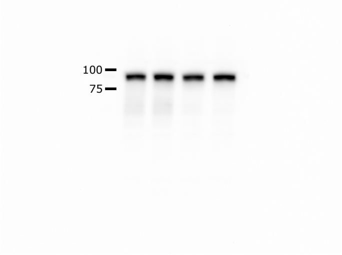

Fig 4H: Vinculin

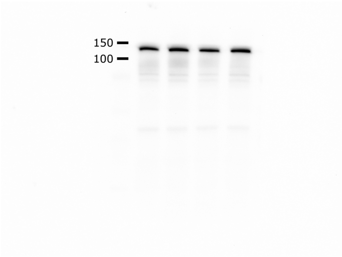

Fig 4H: Actin

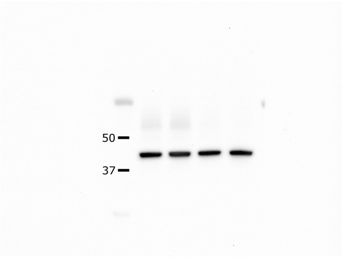

Supplemental Figure S9. Uncropped western blots shown in Figure 5.

Supplemental Figure S9

Uncropped Westerns shown in Figure 5

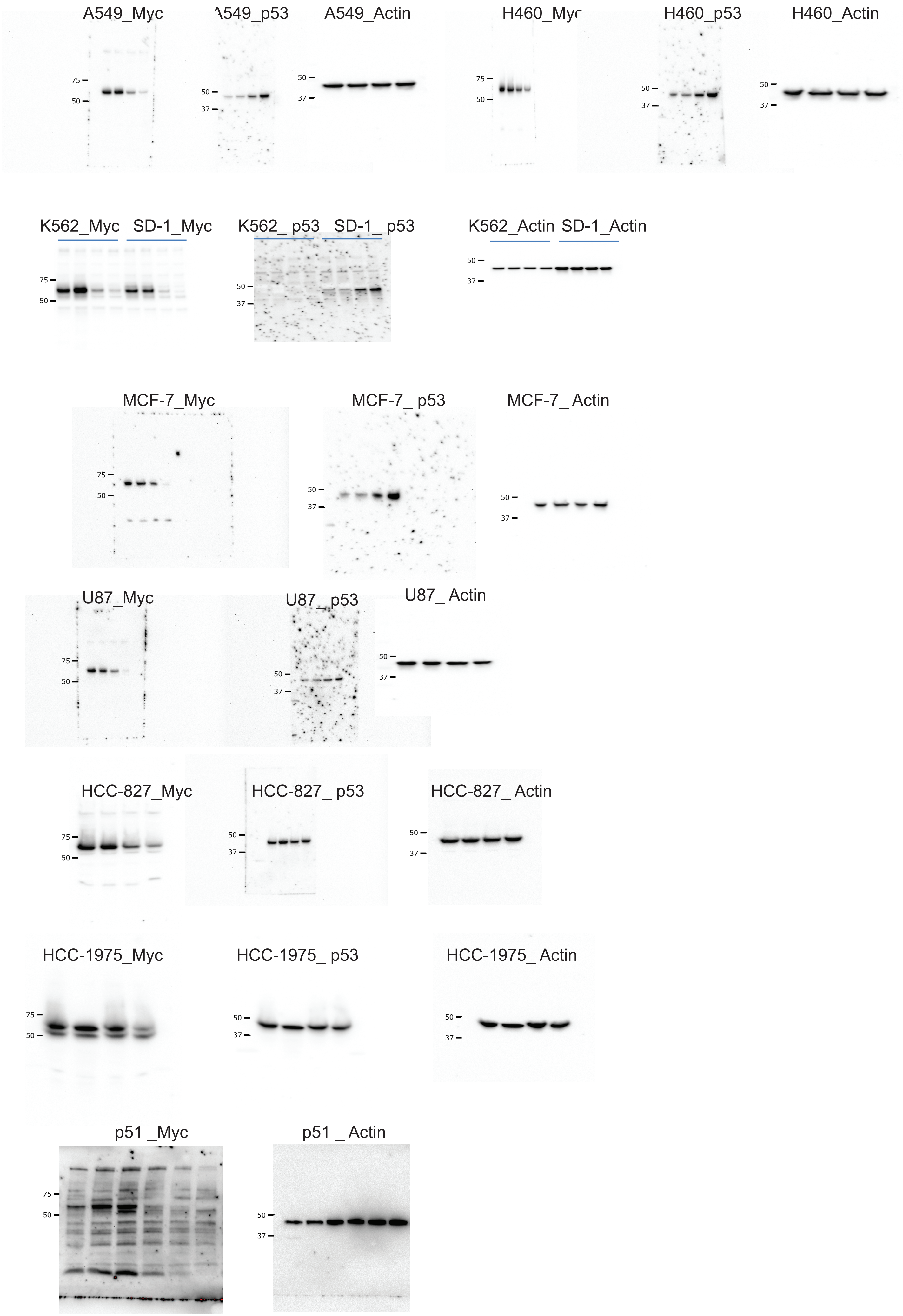

Supplemental Figure S10. Uncropped western blots shown in Figures 6 and 7.

Supplemental Figure S10

Uncropped Westerns shown in Figures 6 and 7

Figure 6C

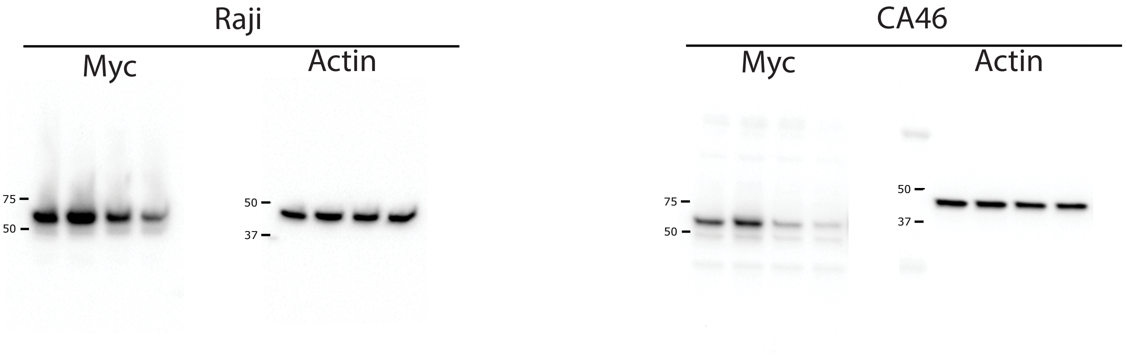

Fig 7B - K562 - c-Myc

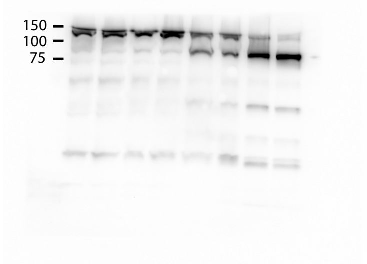

Fig 7B - MV4-11 - c-Myc

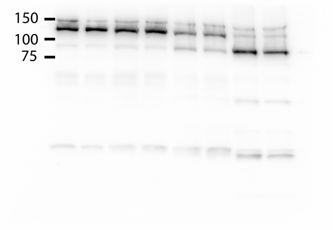

Fig 7B - K562 - Beta-Actin

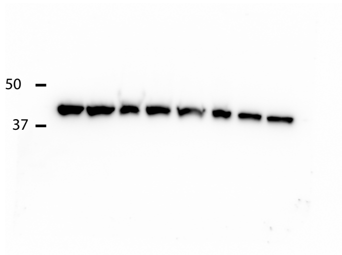

Fig 7B - MV4-11 - Beta-Actin

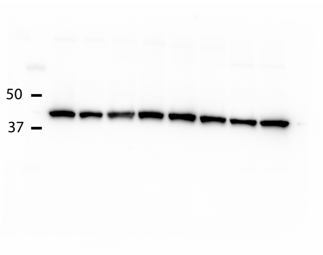

Supplemental Figure S11. Uncropped western blots shown in Suppl. Figure S5.

Supplemental Figure S11

Uncropped Westerns Shown in Suppl. Fig. S5

Fig S5B: Akt pS473

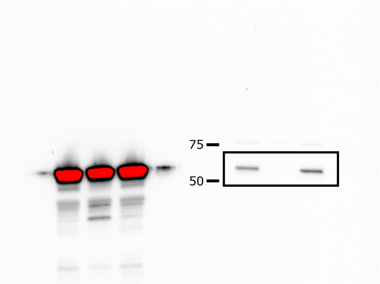

Fig S5B: Akt

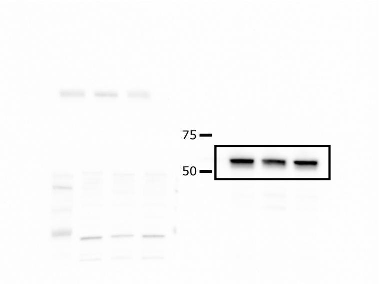

Fig S5B: Stat5 pY694

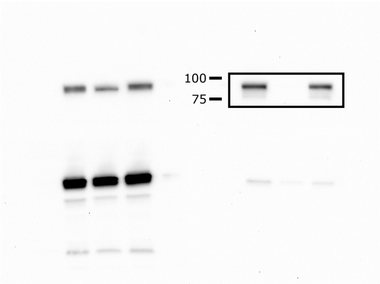

Fig S5B: Stat5

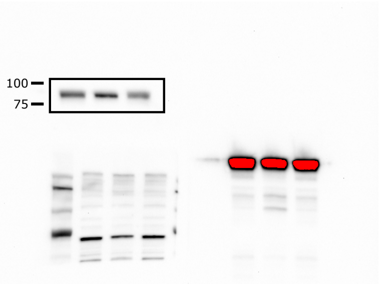

Supplemental Figure S12. Uncropped western blots shown in Suppl. Figure S7.

Supplemental Figure S12

Uncropped Westerns shown in Suppl. Fig. S7

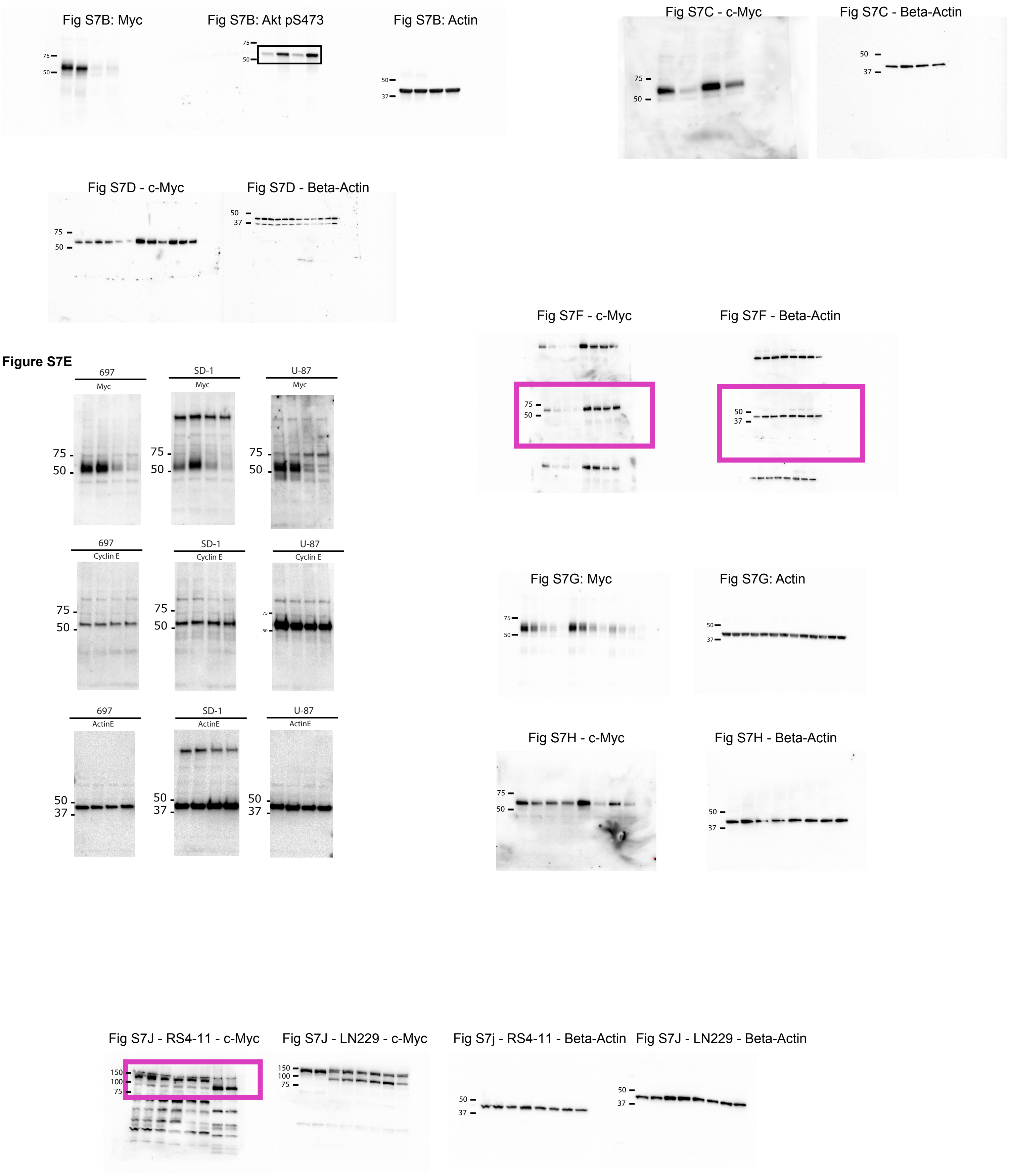

Supplement: Figures S1–S12 [file mmc1.pdf]
